# Morphology and multigene phylogeny reveal five new species and two new records of saprobic microfungi from Yunnan, China

**DOI:** 10.3897/mycokeys.140.205151

**Published:** 2026-09-24

**Authors:** Guang-Cong Ren, Wan-Hao Chen, Heng Gui, Zhuang-Yuan Chen, Chun Guo, Qing-Feng Meng, Wei-Yi Tian

**Affiliations:** 1 School of Pharmacy, Guiyang Kangyang University, Guiyang 550081, China School of Pharmacy, Guiyang Kangyang University Guiyang China; 2 Center for Mycomedicine Research, Basic Medical School, Guizhou University of Traditional Chinese Medicine, Guiyang 550025, China Center for Mycomedicine Research, Basic Medical School, Guizhou University of Traditional Chinese Medicine Guiyang China; 3 Key Laboratory of Microbiome and Infectious Disease Prevention & Control in Guizhou Province, Guiyang 550025, China Key Laboratory of Microbiome and Infectious Disease Prevention & Control in Guizhou Province Guiyang China; 4 Department of Economic Plants and Biotechnology, Yunnan Key Laboratory for Wild Plant Resources, Kunming Institute of Botany, Chinese Academy of Sciences, Kunming 650201, China Department of Economic Plants and Biotechnology, Yunnan Key Laboratory for Wild Plant Resources, Kunming Institute of Botany, Chinese Academy of Sciences Kunming China; 5 School of Public Health, Zunyi Medical University, Zunyi 563000, China School of Public Health, Zunyi Medical University Zunyi China

**Keywords:** *
Anastomitrabeculiaceae
*, *
Coryneaceae
*, *
Dictyosporiaceae
*, five new species, *
Lachnaceae
*, *
Sclerococcaceae
*, *
Tubeufiaceae
*, two new records

## Abstract

During an investigation of saprobic microfungi on dead branches in Yunnan Province, China, seven distinct taxa were obtained using single-spore isolation. Based on detailed morphological examination and multigene phylogenetic analyses (maximum likelihood and Bayesian inference) using ribosomal and protein-coding loci, five new species and two new records were identified. The new species are *Anastomitrabeculia yunnanensis*, *Coryneum lincangense*, *C. lancangense*, *Helicoma nixiense*, and *Sclerococcum xiaokongense*; the two new records are *Dictyocheirospora rotunda* and *Proliferodiscus chiangraiensis*, with the latter representing the first documented asexual morph of the species. These taxa belong to four classes of *Ascomycota*: *Dothideomycetes* (*Pleosporales*, *Tubeufiales*), *Eurotiomycetes (Sclerococcales)*, *Leotiomycetes (Helotiales)*, and *Sordariomycetes (Diaporthales)*. Detailed descriptions, illustrations, and phylogenetic analyses are provided for all taxa.

## Introduction

Saprobic microfungi colonizing woody substrates play important roles in forest ecosystems, particularly in the decomposition and recycling of lignocellulosic materials (Sudharsan et al. 2023). Decaying branches and twigs represent a persistent carbon pool in which specialized fungi secrete a wide array of lignocellulolytic enzymes, thereby driving nutrient cycling and shaping soil properties (Lonsdale et al. 2008; Niego et al. 2023). Despite their ecological significance, the global diversity of saprobic microfungi remains grossly underestimated (Afshari et al. 2025; Ren et al. 2026). Current estimates suggest that the fungal kingdom may contain 1.5–12 million species, but approximately 155,000 fungal species have been described, with millions more likely remaining to be named (Wu et al. 2019; Bhunjun et al. 2022). Fungal taxonomists steadily describe approximately 3,000 new species annually, while fungal molecular ecologists frequently detect thousands of unidentifiable “dark fungi” through metagenomic analyses (Hibbett et al. 2025). Although several fungi, including their asexual morphs, have been reported from decaying branches (Ren et al. 2024, 2026; Wanasinghe et al. 2025), most studies have historically focused on leaf litter, freshwater fungi, or large wood-rotting basidiomycetes (Tennakoon et al. 2022; Shen et al. 2025; Samaradiwakara et al. 2025; Tang et al. 2025). Saprobic microfungi on decaying branches and other woody substrates (trunks, twigs) remain conspicuously understudied across most regions.

Yunnan Province in southwestern China is recognized as a global biodiversity hotspot, characterized by complex topography and highly variable climates (Feng and Yang 2018; Wanasinghe et al. 2020; Hongsanan et al. 2023). The province harbors higher seed plant diversity than any other region in China, along with a wealth of endemic woody plants, both of which provide favorable substrates for wood-decaying fungi (He and Zhao 2022). It has been estimated that Yunnan contains approximately 104,000 fungal species, yet only approximately 6,000 have been formally named (Feng and Yang 2018). In recent years, mycological research in Yunnan has intensified dramatically, leading to the discovery of numerous new taxa across a broad spectrum of ecological niches. These studies cover lignicolous freshwater fungi from rivers, plateau lakes, and karst landscapes (Shen et al. 2022, 2024, 2025; Wang et al. 2024a, 2024b; Zhang et al. 2024, 2025), wood-decaying fungi from various forest areas (He and Zhao 2022; Dong et al. 2023; Yang et al. 2023; Ren et al. 2024; Deng et al. 2025), as well as taxa associated with specific hosts or substrates, such as coffee-associated fungi (Chia et al. 2025; Lu et al. 2025), tea endophytes (Tibpromma et al. 2022), bat-associated fungi (Liu et al. 2023b), entomopathogenic fungi (Chen et al. 2022; Tang et al. 2023), nematode-trapping fungi (Zhang et al. 2022), arbuscular mycorrhizal fungi (Jing et al. 2022; Yuzana et al. 2025), bamboo fungi (Yu et al. 2023), and a large number of new edible mushroom species (Tang et al. 2025). Furthermore, taxonomic and phylogenetic studies continue to produce new genera, new species, and new records, with a particular focus on various families within *Dothideomycetes* and *Sordariomycetes* (Ren et al. 2026; Wang et al. 2026; Xu et al. 2026; Zhou et al. 2026). However, despite these discoveries, the documented species still represent only a tiny fraction of Yunnan’s estimated fungal diversity. This vast gap between described and predicted species underscores the need for continued, systematic surveys to uncover the many unknown saprobic microfungi that remain hidden in this region.

In this study, extensive field surveys were conducted at several locations in Yunnan Province, including Kunming, Lincang, Lancang, Nixi, and Xishuangbanna. Decaying branches were collected, and saprobic microfungi were obtained by spore germination and pure culture isolation. The aims of this paper are to (1) identify and describe five new species and two new records based on detailed morphological examination and multigene phylogenetic analyses of the internal transcribed spacer region (ITS), large subunit ribosomal RNA (LSU), small subunit ribosomal RNA (SSU), translation elongation factor 1-alpha (*tef*1-α), and RNA polymerase II second largest subunit (*rpb*2); (2) provide comprehensive microphotographic illustrations and comparative discussions with allied taxa; and (3) update the phylogenetic placements of *Anastomitrabeculia*, *Coryneum*, *Helicoma*, *Sclerococcum*, *Dictyocheirospora*, and *Proliferodiscus* with the new isolates from dead branches in Yunnan.

## Materials and methods

### Sample collection, morphological observation, and isolation

Dead branch samples were collected from mixed forest habitats in Yunnan Province, China, and transported to the laboratory in sterile plastic bags. Specimens were examined under a stereomicroscope (Olympus SZ61, Tokyo, Japan). Microscopic characters were observed and photographed using a Canon EOS 600D (Canon, Tokyo, Japan) digital camera fitted to a Nikon ECLIPSE 80i compound microscope (Nikon, Tokyo, Japan). Measurements of fungal structures were made with Tarosoft® Image Frame Work v.0.9 software and are presented as minimum–maximum ranges with mean values. Photographic plates were assembled and edited using Adobe Photoshop CS6 (Adobe Systems, San Jose, CA, USA). Pure cultures were established by single-spore isolation following the protocol outlined by Senanayake et al. (2020). Voucher specimens were deposited in the Herbarium of Cryptogams, Kunming Institute of Botany, Chinese Academy of Sciences (HKAS), while living cultures were preserved in the Kunming Institute of Botany Culture Collection (KUNCC), Kunming, China. Faces of Fungi and Index Fungorum numbers were obtained for all newly introduced taxa (Jayasiri et al. 2015; Index Fungorum 2026).

### DNA extraction, PCR amplification, and sequencing

Genomic DNA was extracted from fresh mycelia grown on potato dextrose agar (PDA) at 25 °C for approximately four weeks using the Biospin Fungus Genomic DNA Extraction Kit (BioFlux®, Hangzhou, China), following the manufacturer’s instructions. Five loci, ITS, LSU, SSU, *tef*1-α, and *rpb*2, were amplified using the primer pairs ITS5/ITS4 (White et al. 1990), LR0R/LR5 (Vilgalys and Hester 1990), NS1/NS4 (White et al. 1990), 983F/2218R (Rehner and Buckley 2005), and fRPB2-5F/fRPB2-7cR (Liu et al. 1999), respectively. PCR amplification protocols for SSU, LSU, ITS, *tef*1-α, and *rpb*2 followed Wanasinghe et al. (2021). Amplified products were verified by 1% agarose gel electrophoresis stained with ethidium bromide and subsequently purified and sequenced by Qingke Biotechnology Co., Ltd. (Kunming, Yunnan, China). Newly generated sequences were deposited in GenBank.

### Phylogenetic analyses

Preliminary taxonomic placement of the obtained strains was assessed using BLASTn searches against sequences available in GenBank. Related taxa and representative sequences were selected based on BLAST results and recent literature. Newly generated sequences were assembled and edited in BioEdit v.7.2.3 (Hall 1999). Individual gene datasets were aligned separately using the MAFFT v.7 online server (Katoh et al. 2019) and manually refined where necessary to remove ambiguous regions and excessive gaps. The aligned loci were concatenated in BioEdit for subsequent phylogenetic analyses.

Maximum likelihood (ML) analyses were conducted using RAxML-HPC2 on XSEDE v.8.2.12 (Stamatakis 2014), implemented through the CIPRES Science Gateway (Miller et al. 2010), under the GTRGAMMA substitution model with 1,000 bootstrap replicates. Bayesian inference (BI) analyses were performed in MrBayes v.3.2.7a (Ronquist et al. 2012). The best-fit nucleotide substitution models for each gene partition were estimated using MrModeltest v.2.3 implemented in PAUP v.4.0b10. Six Markov chains were run simultaneously for 2,000,000 generations, with trees sampled every 200 generations. The analysis was terminated when the average standard deviation of split frequencies fell below 0.01. Resulting phylogenetic trees were visualized in FigTree v.1.4.0 (Rambaut 2012) and edited using Adobe Illustrator CS6 and Microsoft PowerPoint.

## Results

### Taxonomy and phylogeny analyses

Integrative taxonomic analyses based on morphological characteristics and multigene phylogenetic data resulted in the identification of seven taxa obtained from decaying branches in Yunnan Province, China. These include five novel species and two new records. Detailed taxonomic descriptions, illustrations, and phylogenetic placements of all identified taxa are provided below. Taxonomic classification follows Hyde et al. (2024a) and Thiyagaraja et al. (2025).

#### *Dothideomycetes* O.E. Erikss & Winka

***Pleosporales* Luttr. ex M.E. Barr**

***Anastomitrabeculiaceae* Bhunjun, Phukhams. & K.D. Hyde**

##### Anastomitrabeculia

Taxon classificationFungiPleosporalesAnastomitrabeculiaceae

Bhunjun, Phukhams. & K.D. Hyde, J. Fungi 7: 12 (2021)

6DF17C9F-876D-5FF5-A50F-E16320C51CDB

###### Notes.

*Anastomitrabeculia* was introduced by Bhunjun et al. (2021), with *A. didymospora* as the type species. Currently, two species are listed in Index Fungorum (2026). *Anastomitrabeculia didymospora* was described as saprobic on dead bamboo culms submerged in freshwater based on its sexual morph (Bhunjun et al. 2021), whereas *A. xishuangbannaensis* was reported from dead woody twigs of *Knema furfuracea* in terrestrial habitats based on its asexual morph (Ren et al. 2024). The sexual morph is characterized by semi-immersed, coriaceous or carbonaceous ascomata with septate, trabeculate pseudoparaphyses, and hyaline ascospores with longitudinally striate wall ornamentation, surrounded by a mucilaginous sheath (Bhunjun et al. 2021). The asexual morph is coelomycetous, with immersed, ostiolate conidiomata bearing phialidic conidiogenous cells and dictyosporous, muriform conidia that are oval to obovoid, hyaline when young, and brown to dark brown at maturity (Ren et al. 2024). In this study, a new species of *Anastomitrabeculia* from China is introduced based on its sexual morph.

##### Anastomitrabeculia yunnanensis

Taxon classificationFungiPleosporalesAnastomitrabeculiaceae

G.C. Ren & W.Y. Tian sp. nov.

47C99CC1-0538-5B2E-9773-ECC5347F4205

Index Fungorum: IF905583

Facesoffungi Number: FoF20021

Fig. 2

###### Etymology.

The species epithet “*yunnanensis*” refers to the province where the species was collected.

###### Holotype.

HKAS 122775

###### Description.

***Saprobic*** on decaying branches in terrestrial habitats. **Sexual morph: *Ascomata*** 520–860 μm high, 290–470 μm diam. (x̄ = 730 × 400 μm µm, *n* = 5), clustered, immersed to erumpent through the host periderm, visible as raised, subglobose, uni-loculate, coriaceous, black, ostiolate. ***Ostioles*** central, 124–257 µm high, 81–128 µm diam. (x̄ = 197 × 106 µm, *n* = 5). ***Peridium*** 23–36 µm wide, composed of several layers of flattened cells, outer cells, darker, fusing and indistinguishable from the host tissues, inner cells hyaline, thin-walled, arranged in a *textura angularis*. ***Hamathecium*** 2–4 µm wide, comprising numerous, branched, cylindrical to filamentous, septate, hyaline pseudoparaphyses. ***Asci*** 226–420 × 40–47 µm (x̄ = 290 × 44 µm, *n* = 20), 6–8-spored, bitunicate, fissitunicate, elongate-clavate, with a long pedicel (115–190 µm long; x̄ = 140 μm, *n* = 10). ***Ascospores*** 36.6–45.7 × 13.5–21.8 µm (x̄ = 40.4 × 18.3 µm, *n* = 40), overlapping uni- to bi-seriate, muriform, ellipsoidal to broadly fusiform, 2-celled, multi-septate, sectored, hyaline to pale brown when young, becoming brown at maturity, constricted at the central septum, upper part larger than the lower part, guttulate in each cell, ends remaining cone-shaped, with acute ends, surrounded by mucilaginous sheath. **Asexual morph**: Undetermined.

###### Culture characteristics.

Ascospores germinate on PDA within 24 h at room temperature. Germ tubes are produced from every cell of the spore. Colonies on PDA reach 50 mm in diameter after 2 weeks at 20–25 °C; mycelium superficial and circular; surface rough, with sparse, grayish-white, floccose, flat mycelium; reverse grayish white with gray spots.

###### Material examined.

China • Yunnan Province, Xishuangbanna, on decaying branches of *Quercus variabilis* (*Fagaceae*), 15 December 2019, G.C. Ren, XS20 (HKAS 122775, holotype), ex-type living culture KUNCC 21-0673.

###### GenBank numbers.

ITS: PZ457828; LSU: PZ454096; SSU: PZ487682; *tef*1-α: PZ480917.

###### Notes.

**Figure 1.**
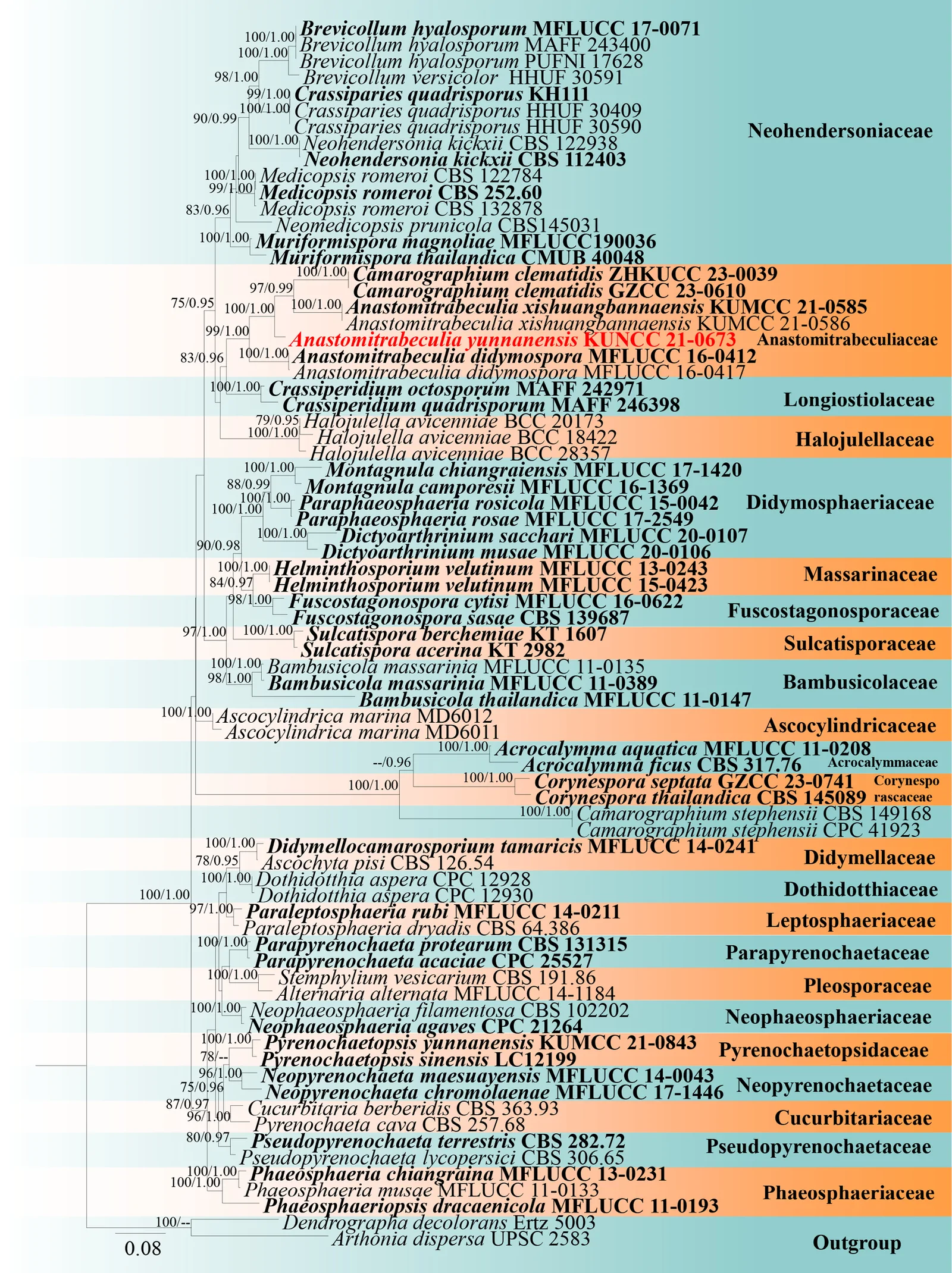
Phylogram generated from ML analysis based on SSU, LSU, ITS, and *tef*1-α sequence data. Related sequences were obtained following Ren et al. (2024) and BLAST search results in GenBank. Seventy-five strains are included in the combined analyses, which comprise 3,198 characters for the SSU, LSU, ITS, and *tef*1-α alignment. *Arthonia dispersa* (UPSC 2583) and *Dendrographa decolorans* (Ertz 5003) were used as the outgroup taxa. The best-scoring RAxML tree with a final likelihood value of –28,027.766070 is presented. The matrix had 1,449 distinct alignment patterns, with 27.23% undetermined characters or gaps. Estimated base frequencies were as follows: A = 0.243048, C = 0.240167, G = 0.272106, T = 0.244679. Substitution rates were AC = 1.289576, AG = 2.841996, AT = 1.802252, CG = 0.832289, CT = 6.579166, and GT = 1.0000. Gamma distribution shape parameter *α* = 0.269377, and tree length = 4.396201. The tree topology of the ML analysis is similar to that of the Bayesian analysis. Bootstrap values for ML equal to or greater than 70% and posterior probability values greater than 0.95 from the BYPP analysis are labeled at the nodes. Strains of the newly described species are in red, while type strains are in bold.

Multilocus phylogenetic analyses based on a concatenated SSU, LSU, ITS, and *tef*1-α sequence dataset show that the strain KUNCC 21-0673 clusters with *Anastomitrabeculia* species with 100% ML bootstrap support and 1.00 BYPP (Fig. 1). Morphologically, *Anastomitrabeculia yunnanensis* (HKAS 122775, holotype) is distinguished from *A. didymospora* (MFLU 20-0694, holotype) by having larger asci (226–420 × 40–47 μm vs. 125–160 × 15–20 μm) with a long pedicel and muriform, ellipsoidal to broadly fusiform, brown ascospores, while *A. didymospora* has broadly fusiform and hyaline ascospores (Bhunjun et al. 2021). *Anastomitrabeculia xishuangbannaensis* was introduced from its asexual morph (Ren et al. 2024), whereas *A. yunnanensis* is introduced here from its sexual morph. Therefore, a morphological comparison between the new species and *A. xishuangbannaensis* was not possible. Sequence comparisons between *Anastomitrabeculia yunnanensis* (KUMCC 21-0673) and *A. didymospora* (MFLUCC 16-0412) showed base-pair differences of 12.1% (55/453 bp, without gaps) in the ITS region, 7.6% (62/815 bp, without gaps) in the *tef*1-α region, and 3.7% (31/835 bp, without gaps) in the LSU region. Additionally, sequence comparisons between *Anastomitrabeculia yunnanensis* (KUMCC 21-0673) and *A. xishuangbannaensis* (KUMCC 21-0585) showed base-pair differences of 9.5% (42/444 bp, without gaps) in the ITS region, 12.4% (107/861 bp, without gaps) in the *tef*1-α region, and 3.2% (26/808 bp, without gaps) in the LSU region. Based on both morphological distinctions and its well-supported phylogenetic placement, *Anastomitrabeculia yunnanensis* is introduced as a novel species.

**Figure 2.**
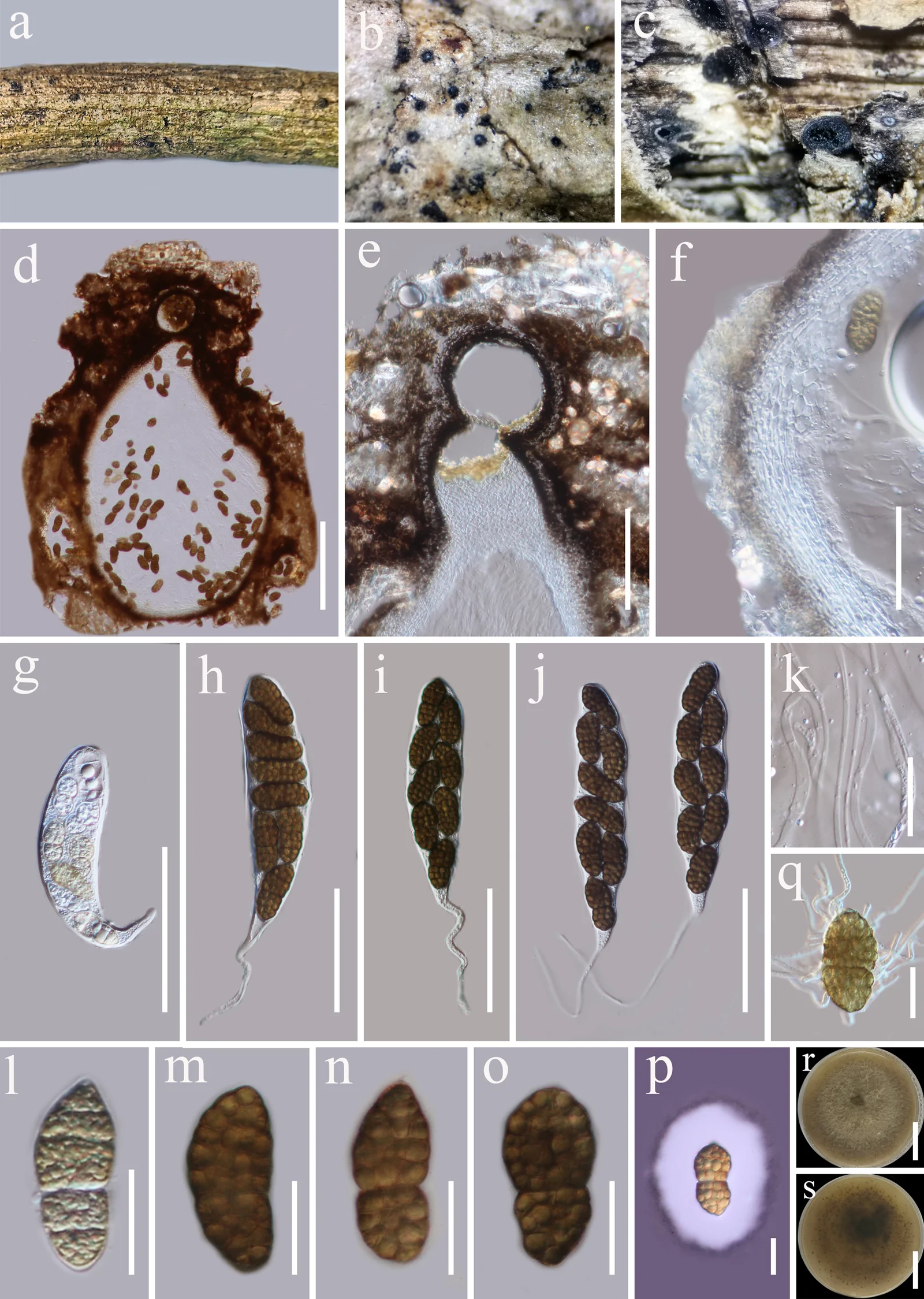
*Anastomitrabeculia yunnanensis* (HKAS 122775, holotype). **a**. Herbarium specimen; **b**. Ascomata on substrate; **c**. Ascomata in transverse section; **d**. Section of an ascoma; **e**. Section of ostiole; **f**. Peridium; **g–j**. Asci; **k**. Pseudoparaphyses; **l–o**. Ascospores; **p**. An ascospore with a mucilaginous sheath in Indian ink; **q**. A germinating ascospore; **r, s**. Colony on PDA, surface, and reverse views. Scale bars: 200 μm (**d**); 100 μm (**e, g–j**); 50 μm (**f, l–q**); 20 μm (**k**); 30 mm (**r, s**).

#### *Dictyosporiaceae* Boonmee & K.D. Hyde

##### Dictyocheirospora

Taxon classificationFungiPleosporalesDictyosporiaceae

M.J. D’souza, Boonmee & K.D. Hyde, Fungal Diversity 80: 465 (2016)

04D5DE97-1DC0-51E8-95EC-F467A583CF8B

###### Notes.

*Dictyocheirospora* was introduced by Boonmee et al. (2016) with *D. rotunda* as the type species. The genus is mainly known from its asexual morphs, which are characterized by dark sporodochial colonies that produce cheiroid, non-complanate, ellipsoid to cylindrical conidia (Boonmee et al. 2016; Liu et al. 2023a). Currently, 36 species are accepted in *Dictyocheirospora* (Index Fungorum 2026); members of the genus are commonly reported as saprobes on woody litter in both terrestrial and freshwater habitats (Shen et al. 2025).

##### Dictyocheirospora rotunda

Taxon classificationFungiPleosporalesDictyosporiaceae

D’souza, Bhat & K.D. Hyde, Fungal Diversity, 80: 465 (2016)

C1EB42DE-ECD9-5134-90FC-2612E13353A5

Index Fungorum: IF551581

Facesoffungi Number: FoF01262

Fig. 4

###### Description.

***Saprobic*** on decaying branches in terrestrial habitats. **Asexual morph**: Hyphomycetous. Colonies on natural substrates scattered, sporodochial, brown to dark brown. ***Mycelium*** immersed, composed of branched, septate, pale brown, smooth-walled hyphae. ***Conidiophores*** micronematous, mononematous, septate, cylindrical, hyaline to pale brown, smooth-walled, mostly reduced to conidiogenous cells. ***Conidiogenous cells*** 4–17 × 3–5 μm (x̄ = 9 × 4.5 μm, *n* = 20), holoblastic, cylindrical, hyaline to pale brown, smooth-walled. ***Conidia*** 42–48 × (15–)18–24 μm (x̄ = 45 × 21 μm, *n* = 50), solitary, cheiroid, ellipsoid to cylindrical, with a basal connecting cell, pale brown to brown, composed of 5–7 rows of cells, euseptate, unseparated, each row with 10–12 cells, guttulate, slightly constricted at septa, without appendages. **Sexual morph**: Undetermined.

###### Culture characteristics.

Conidia germinate on PDA within 24 h at room temperature. Germ tubes are produced at the basal region. Colonies on PDA reach 50 mm in diameter after 2 weeks at 20–25 °C; mycelium superficial and circular; surface rough, raised at the center, zonate, grayish white, floccose, and flat; reverse light pink and zonate.

###### Material examined.

China • Yunnan Province, Kunming, Xiaokong, on decaying branches of *Betula albosinensis* (*Betulaceae*), 21 December 2019, G.C. Ren, KM15 (HKAS 122746), living culture KUNCC 21-0596.

###### Known distribution and hosts.

China and Thailand, on decaying wood from freshwater habitats (Boonmee et al. 2016; Wang et al. 2016; Liu et al. 2023a; Shen et al. 2025) and on decaying branches of *Betula albosinensis* (*Betulaceae*) in terrestrial habitats (this study).

###### GenBank numbers.

ITS: PZ457829; LSU: PZ454097; SSU: PZ487683; *tef*1-α: PZ480918; *rpb*2: PZ480923.

###### Notes.

**Figure 3.**
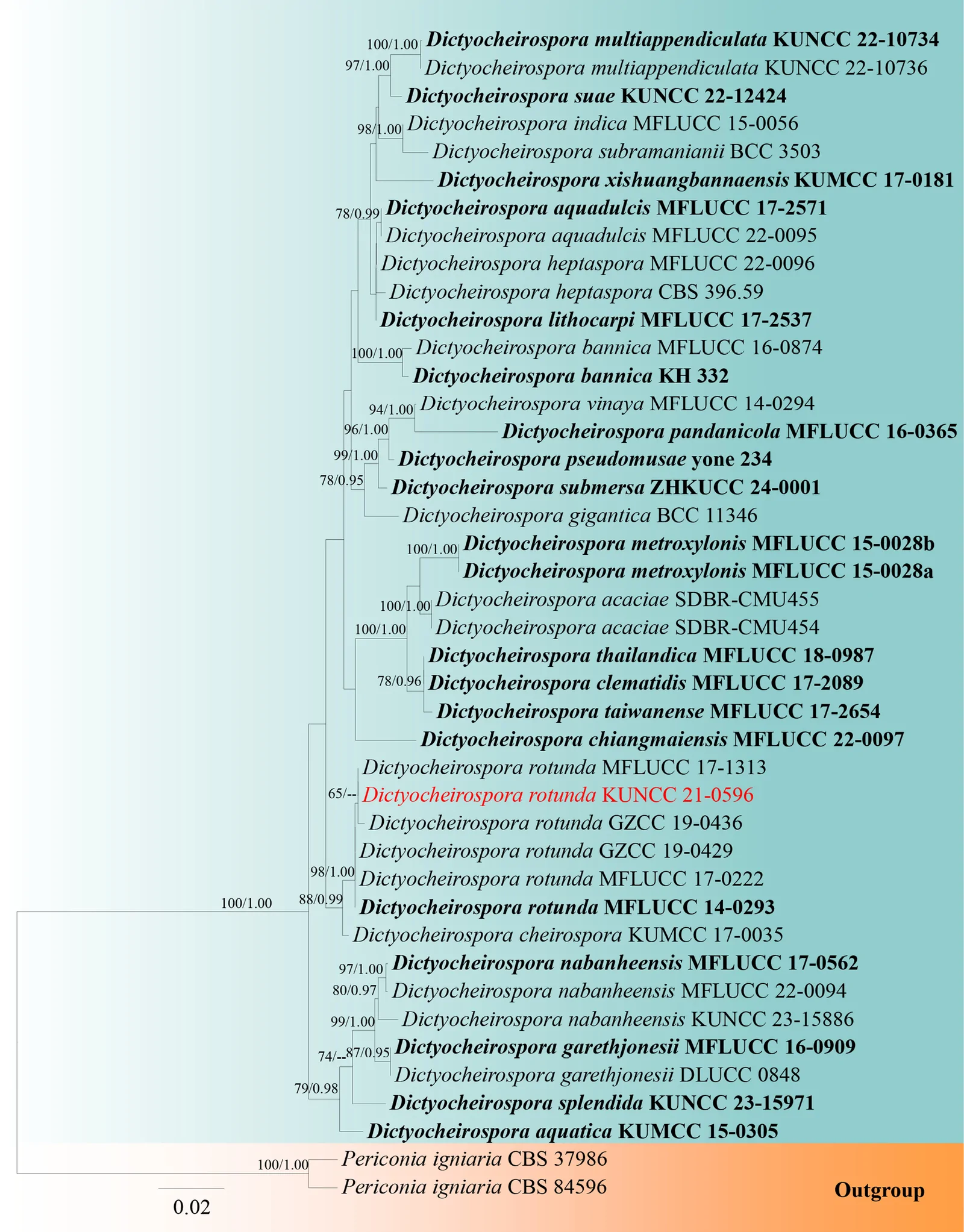
Phylogram generated from ML analysis based on LSU, ITS, and *tef*1-α sequence data. Related sequences were obtained following Wang et al. (2025). Forty-two strains are included in the combined analyses, which comprise 2,288 characters for the LSU, ITS, and *tef*1-α alignment. *Periconia igniaria* (CBS 379.86 and CBS 845.96) was used as the outgroup taxon. The best-scoring RAxML tree with a final likelihood value of –7,612.799553 is presented. The matrix had 509 distinct alignment patterns, with 19.12% undetermined characters or gaps. Estimated base frequencies were as follows: A = 0.234612, C = 0.263458, G = 0.269132, T = 0.232798. Substitution rates were AC = 2.026548, AG = 3.320387, AT = 2.462825, CG = 1.011355, CT = 10.840798, and GT = 1.0000. Gamma distribution shape parameter *α* = 0.233098 and tree length = 0.479556. The tree topology of the ML analysis is similar to that of the Bayesian analysis. Bootstrap values for ML equal to or greater than 70% and posterior probability values greater than 0.95 from the BYPP analysis are labeled at the nodes. Strains of the newly described species are in red, while type strains are in bold.

*Dictyocheirospora rotunda*, the type species of *Dictyocheirospora*, was introduced by Boonmee et al. (2016) from decaying wood in freshwater habitats. In the multigene phylogenetic analyses based on a combined LSU, ITS, and *tef*1-α dataset, the new isolate KUNCC 21-0596 clustered with reference strains of *Dictyocheirospora rotunda* (MFLUCC 14-0293, MFLUCC 17-0222, GZCC 19-0436, GZCC 19-0429, MFLUCC 17-1313) (Fig. 3). Morphologically, the fresh collection (HKAS 122746) is fully consistent with the original description of *Dictyocheirospora rotunda* (MFLU 14-0253, holotype) (Boonmee et al. 2016; Shen et al. 2025). Sequence comparisons of the LSU, ITS, and *tef*1-α regions between the isolate (KUNCC 21-0596) and *Dictyocheirospora rotunda* (MFLUCC 14-0293) revealed no significant nucleotide differences. The collection is therefore identified as *Dictyocheirospora rotunda*, representing a new host record from *Betula albosinensis* (*Betulaceae*) in terrestrial habitats in China.

**Figure 4.**
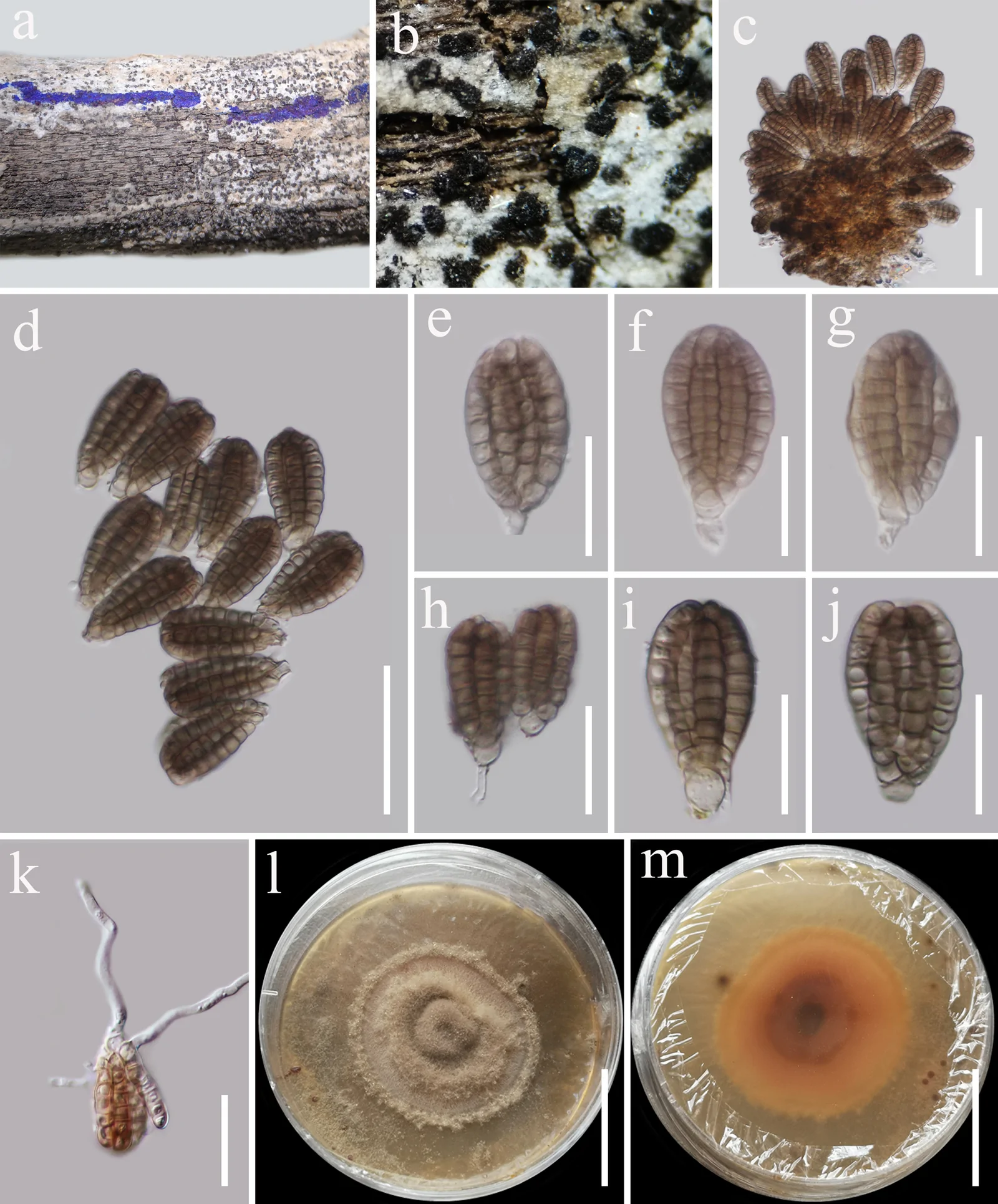
*Dictyocheirospora rotunda* (HKAS 122746). **a, b**. Colonies on natural substrate; **c**. Conidiogenous cells and conidia; **d–j**. Conidia; **h**. Germinated conidium; **l, m**. Colony on PDA, surface, and reverse views. Scale bars: 50 μm (**c, d**); 30 μm (**e–k**); 20 mm (**l, m**).

#### *Tubeufiales* Boonmee, Rossman & K.D. Hyde

***Tubeufiaceae* M.E. Barr**

##### Helicoma

Taxon classificationFungiTubeufialesTubeufiaceae

Corda, Icones fungorum hucusque cognitorum1: 15 (1837)

A05B0EDE-C519-52E5-9D59-6E12734F768C

###### Notes.

*Helicoma* was established by Corda (1837), with *H. muelleri* as the type species. The genus has a worldwide distribution and occurs in both freshwater and terrestrial habitats (Lu et al. 2018, 2023). Species of *Helicoma* are commonly reported as saprobes. Currently, 77 species are included in *Helicoma* (Ma et al. 2024; Xiong et al. 2024; Karimi et al. 2025; Xiao et al. 2025). Most *Helicoma* species have been described based on their asexual morphological characters, while only six sexual species have been introduced with supporting molecular data (Boonmee et al. 2014; Lu et al. 2018). In this study, a new sexual species collected from woody litter in China is introduced.

##### Helicoma nixiense

Taxon classificationFungiTubeufialesTubeufiaceae

G.C Ren & W.Y Tian sp. nov.

0304C486-A3C2-5616-91E0-5965F3ACEAB0

Index Fungorum: IF905584

Facesoffungi Number: FoF20022

Fig. 6

###### Etymology.

The specific epithet “*nixiense*” refers to Nixi, where the holotype was collected.

###### Holotype.

HKAS 122761.

###### Description.

***Saprobic*** on decaying branches in terrestrial habitats. **Sexual morph: *Ascomata*** 260–320 μm high × 159–260 μm diam. (x̄ = 280 × 200 μm, *n* = 5), superficial, solitary or clustered, gregarious, uni-loculate, oval to obovoid, with soft texture, dark brown, ostiole. ***Peridium*** 20–45 μm wide, composed of dark brown cells of ***textura angularis***. *Hamathecium* 1–2 μm diam., rarely, filiform, septate, branched, hyaline pseudoparaphyses. ***Asci*** 157–210 × 26–44 μm (x̄ = 190 × 35 μm, *n* = 20), 8-spored, bitunicate, clavate to broadly clavate, with a short pedicel, rounded to truncate at the apex, thickened at apex, ocular chamber not observed. ***Ascospores*** 94–107 × 6.8–11.7 μm (x̄ = 100 × 9.6 μm, *n* = 30), 2–3-seriate, elongate-fusiform, subcylindrical, tapering towards subacute ends, 13–17-septate, not constricted at septum, hyaline, smooth-walled. **Asexual morph**: Undetermined.

###### Culture characteristics.

Ascospores germinate on PDA within 24 h at room temperature. Germ tubes are produced from the basal or apical cell of the ascospore. Colonies on PDA reach 20 mm in diameter after 2 weeks at 20–25 °C; growth slow; mycelium superficial, raised, circular, fimbriate, dense, flat, with an entire edge, dark brown; reverse dark brown.

###### Material examined.

China • Yunnan Province, Diqing, Xianggelila, Nixi, (27°25'37"N, 99°50'45"E, elevation: 3,090 m), on decaying branches of *Pellacalyx yunnanensis* (*Rhizophoraceae*), 31 August 2020, G.C. Ren, NX04 (holotype, HKAS 122761), ex-type culture KUNCC 21-0638.

###### GenBank numbers.

ITS: PZ457830; LSU: PZ454098; SSU: PZ487684; *tef*1-α: PZ480919.

###### Notes.

**Figure 5.**
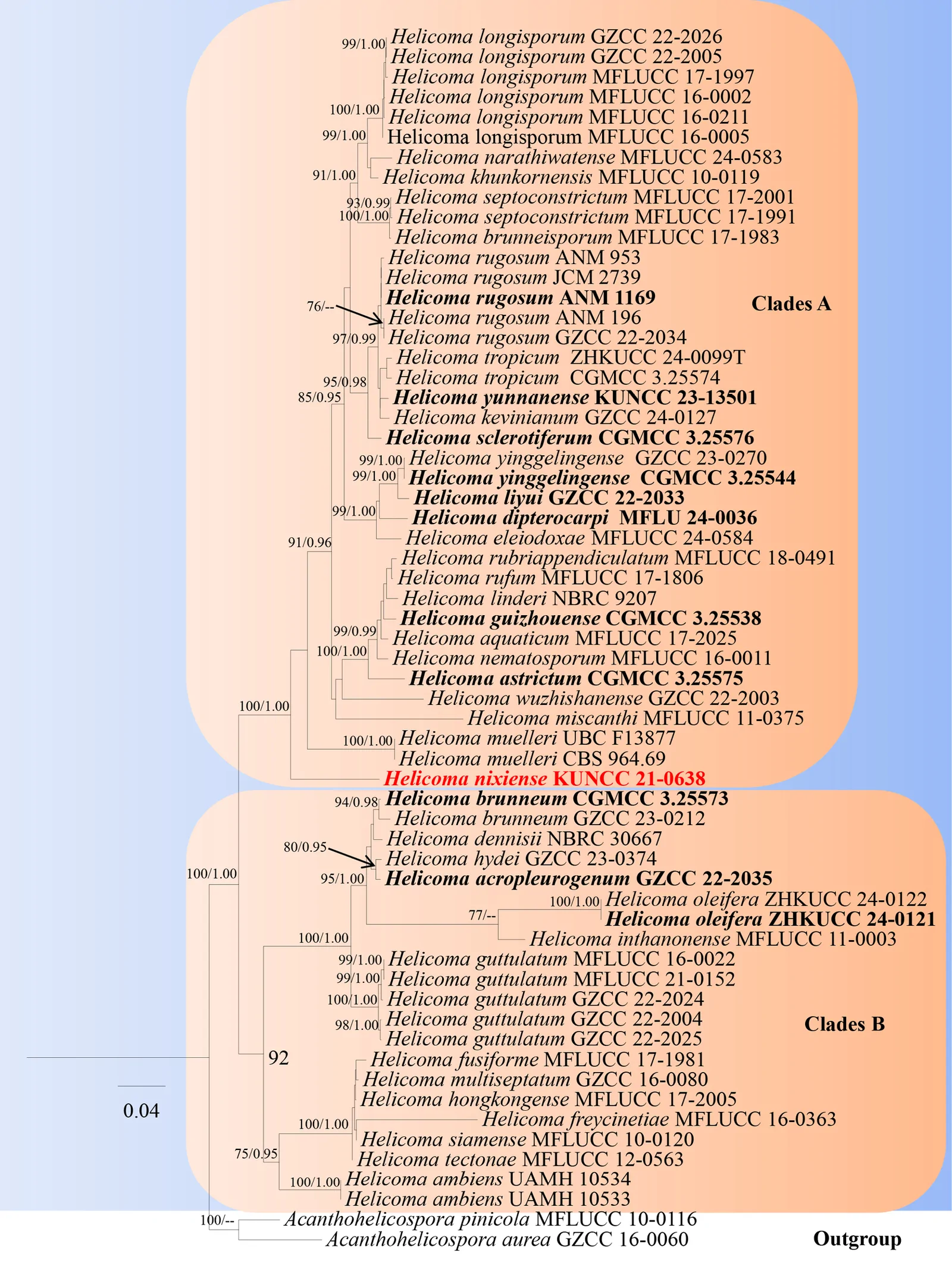
Phylogram generated from ML analysis based on LSU, ITS, *tef*1-α, and *rpb*2 sequence data. Related sequences were obtained following Lu et al. (2022) and Karimi et al. (2025). Sixty-one strains are included in the combined analyses, which comprise 3,371 characters for the LSU, ITS, *tef*1-α, and *rpb*2 alignment. *Acanthohelicospora aurea* (GZCC 16-0060) and *A. pinicola* (MFLUCC 10-0116) were used as the outgroup taxa. The best-scoring RAxML tree with a final likelihood value of –19,867.964128 is presented. The matrix had 1,235 distinct alignment patterns, with 25.18% undetermined characters or gaps. Estimated base frequencies were as follows: A = 0.249703, C = 0.239778, G = 0.253573, T = 0.256946. Substitution rates were AC = 1.297377, AG = 4.321841, AT = 2.022859, CG = 0.885859, CT = 9.320627, and GT = 1.0000. Gamma distribution shape parameter *α* = 0.234399 and tree length = 1.604913. The tree topology of the ML analysis is similar to that of the Bayesian analysis. Bootstrap values for ML equal to or greater than 70% and posterior probability values greater than 0.95 from the BYPP analysis are labeled at the nodes. Strains of the newly described species are in red, while type strains are in bold.

Multigene phylogenetic analyses based on a concatenated LSU, ITS, *tef*1-α, and *rpb*2 dataset recovered two major monophyletic clades within *Helicoma* (Clades A and B; Fig. 5). The new collection (KUNCC 21-0638) formed a distinct lineage basal to Clade A, receiving strong statistical support (100% ML/1.00 BYPP; Fig. 5). Morphologically, *Helicoma nixiense* (HKAS 122761, holotype) resembles other *Helicoma* species in having superficial, oval to obovoid ascomata, a *textura angularis* peridium, cylindric-clavate asci with a thickened apex, and 2–3-seriate, elongate-fusiform to subcylindrical ascospores tapering toward subacute ends (Boonmee et al. 2014; Lu et al. 2018). However, *Helicoma nixiense* (HKAS 122761, holotype) can be clearly distinguished from the other six *Helicoma* species (*H. fusiforme*, *H. miscanthi*, *H. rugosa*, *H. siamense*, *H. chiangraiense*, and *H. fagacearum*) by its unique combination of sexual morphological characters, particularly in ascus and ascospore dimensions (Table 1). The asci of *H. nixiense* (HKAS 122761, holotype) (157–210 × 26–44 µm) are clavate to broadly clavate and are generally larger and broader than those of *H. fusiforme* (MFLU 17-1123, holotype) and *H. miscanthi* (MFLU 11-0134, epitype). The remaining species, *H. rugosa* (BPI 1104599), *H. siamense* (MFLU 10-0053, holotype), *H. chiangraiense* (MFLU 10-0048, holotype), and *H. fagacearum* (MFLU 11-0138, holotype), also possess comparatively smaller and more slender asci (Boonmee et al. 2014; Lu et al. 2018; Table 1). The ascospores of *H. nixiense* (HKAS 122761, holotype) (94–107 × 6.8–11.7 µm, 13–17-septate) are considerably larger and more septate than those of *H. chiangraiense* (MFLU 10-0048, holotype), *H. fusiforme* (MFLU 17-1123, holotype), *H. rugosa* (BPI 1104599), *H. siamense* (MFLU 10-0053, holotype), and *H. fagacearum* (MFLU 11-0138, holotype) (Boonmee et al. 2014; Lu et al. 2018; Table 1), while *H. miscanthi* (MFLU 11-0134, epitype) has larger and more septate ascospores than *H. nixiense* (HKAS 122761, holotype) (Lu et al. 2018; Table 1). Additionally, *H. nixiense* (HKAS 122761, holotype) typically lacks prominent ostiolar setae, whereas *H. rugosa* (BPI 1104599) uniquely exhibits conspicuous setae (Boonmee et al. 2014). Based on both morphological distinctions and its well-supported phylogenetic placement, *Helicoma nixiense* is introduced as a novel species.

**Figure 6.**
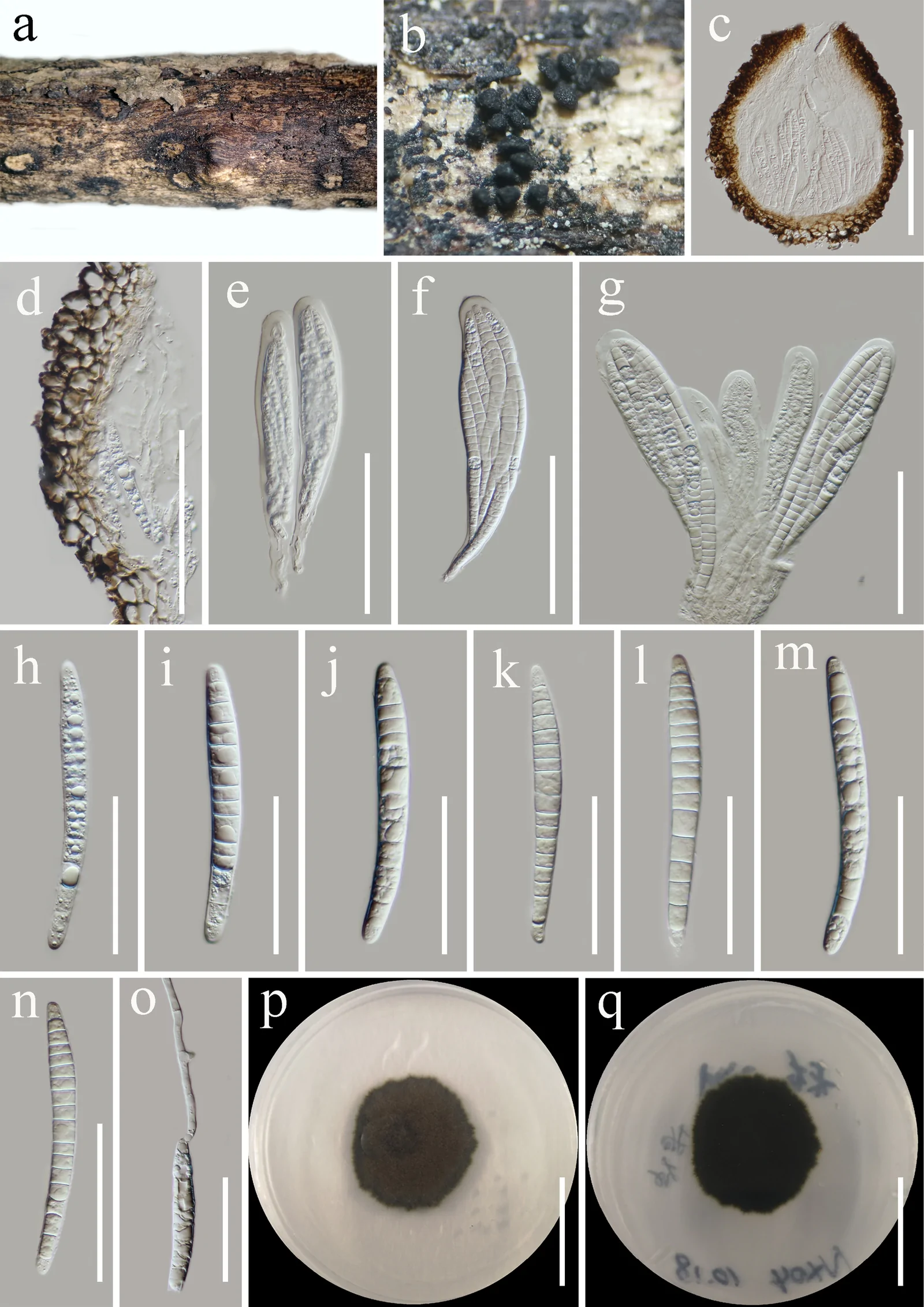
*Helicoma nixiense* (HKAS 122761, holotype). **a**. Herbarium specimen; **b**. Ascomata on substrate; **c**. Cross section of an ascoma; **d**. Peridium; **e–g**. Asci; **h–n**. Ascospores; **o**. Germinating ascospore; **p, q**. Colony on MEA, surface, and reverse views. Scale bars: 150 μm (**c**); 100 μm (**d–g**); 50 μm (**h–o**); 30 mm (**p, q**).

**Table 1.** Morphological comparison of *Helicoma* species (sexual morph).

**Species name**	**Ascomata (µm)**	**Peridium (µm)**	**Asci (µm)**	**Ascospores (µm)**	**Reference**
*Helicoma fusiforme*	210–270 × 250–290, dark brown, coriaceous	30–45, inner cells brown and outer cells dark brown	115–145 × 20–30, cylindrical, short-pedicellate	60–70 × 7–8.5, fusiform, hyaline, 10–11-septate	Lu et al. (2018)
*H. miscanthi*	285–358(–373) × 299–339(–386), brown to dark brown, with a waxy covering on ascomal wall	51–58, brown to red brown cells	(142-)154–188(-194) × 19–26(-30), cylindric-clavate, mostly apedicillate	(122-)131–165(-187.5) × 7–10, cylindric-clavate, elongate, hyaline, 19–26(-30) -septate	Boonmee et al. (2014)
*H. rugosa*	(242-)303–340 × (218-) 238–263(–331), dark brown, soft texture, with rare setae	61–80, brown to reddish-brown cells	(90-)96–135 × 15–20, elongate to cylindric-clavate, long pedicels	42–61 × 5–8.5, elongate-fusiform, subcylindrical, hyaline, (8-)9–10-septate	Boonmee et al. (2014)
*H. siamense*	221–306 × 180–268, reddish-brown to dark brown, with a central ostiole covered by brown mycelium	30–48, outer layer cells darkened and inner layer cells pale brown to hyaline	(123-)127.5–139(-144) × (14-)17–20, cylindrical to clavate, short-pedicellate	(49.5-)59–64.5(-68) × 5–7.5, cylindric-fusiform, hyaline, 11–12-septate	Boonmee et al. (2014)
*H. chiangraiense*	(183-)240–285 × (189-)241–296, dark brown to black, with few hyphae developing from ascomatal	28–34, inner cells brown and outer cells dark brown	(146-)153–179.5 × 18–23(-26), cylindrical, long pedicel	(63-)72–93.5 × 5–7.5, cylindric-fusiform, hyaline, 11–13-septate	Boonmee et al. (2014)
*H. fagacearum*	256–300(–324) × 235–258(–265), yellowish or brownish	43–51, inner cells pale brown and outer cells reddish-brown to brown	(121-)134–160(-179) × 10–14, elongate-calyclindrical, pedicellate	(63-)71–86.5 × 4–6.5, cylindric-fusiform, hyaline, pale brown at maturity, 9–12(-13)-septate	Boonmee et al. (2014)
*H. nixiense*	260–320 × 159–260, dark brown, soft texture,	20–45, dark brown cells	157–210 × 26–44, clavate to broadly clavate, short pedicel	94–107 × 6.8–11.7, elongate-fusiform, subcylindrical, hyaline, 13–17-septate	This study

#### *Eurotiomycetes* O.E. Erikss. & Winka

***Sclerococcales* Réblová, Unter. & W. Gams**

***Sclerococcaceae* Réblová, Unter. & W. Gams, Mycol. Progr. 16(1): 35 (2016) [2017]**

##### Sclerococcum

Taxon classificationFungiTubeufialesSclerococcaceae

Fr., Novitiae florae svecicae 5 (2): 79 (1819)

DCC56F07-DBF4-57C9-8204-DE342B47C342

###### Notes.

*Sclerococcum* was established by Fries (1819, 1825), with *S. sphaerale* as the type species. Subsequent phylogenetic analyses demonstrated that *Dactylospora* represents the sexual morph of *Sclerococcum* (Diederich et al. 2013, 2018). Species of this genus occupy diverse ecological niches, occurring on dead wood and liverworts, or as lichenicolous fungi and helicosporous hyphomycetes in both terrestrial and marine habitats (Diederich et al. 2024; Ma et al. 2024; Meng et al. 2025). In this study, a new species of *Sclerococcum* from a terrestrial habitat in China is introduced.

##### Sclerococcum xiaokongense

Taxon classificationFungiTubeufialesSclerococcaceae

G.C Ren & W.Y Tian sp. nov.

2E62291E-91EA-56A7-9FBC-EF466D56DC0D

Index Fungorum: IF905585

Facesoffungi Number: FoF20023

Fig. 8

###### Etymology.

The specific epithet “*xiaokongense*” refers to Xiaokong, where the holotype was collected.

###### Holotype.

HKAS 122745.

###### Description.

***Saprobic*** on decaying branches in terrestrial habitats. **Sexual morph: *Ascomata*** apothecioid, 235–260 μm high, 600–840 μm diam. (x̄ = 250 × 690 μm, *n* = 5), superficial, rounded, cup-shaped, sessile, arising singly or in small groups, black. ***Disc*** flat, black, finely granulated surface. ***Margin*** Not clearly differentiate from the disc, concolorous to receptacle. ***Exciple*** pseudoparenchymatic, composed of cells with pale to dark brown walls, excipular cells in the marginal parts of the exciple arranged in radiating rows. ***Hymenium*** 66–92 μm high (x̄ = 82 μm, *n* = 30), hyaline to light brown, enclosed in a thick gelatinous matrix. ***Epithecium*** 12–20 μm thick (x̄ = 15.6 μm, *n* = 30), with dark brownish granules. ***Paraphyses*** 1.5–3 μm wide, anastomosing, branched, septate, with swollen, pigmented apices, extending beyond the asci and apices glued together to develop pseudo epithecium. ***Hypothecium*** 125–152 μm thick (x̄ = 138 μm, *n* = 30), pale to dark brown, composed of short-celled interwoven hyphae. ***Asci*** 53–74 × 8.8–12 μm (x̄ = 62.8 × 9.8 μm, *n* = 20), 8-spored, bitunicate, narrowly clavate to cylindrical, short sessile, ascus wall thickened at the apex, ocular chamber absent, rounded at the apex, inoperculate with an amyloid gelatinous cap. ***Ascospores*** 13–19 × 4–4.9 μm (x̄ = 15.6 × 4.4 μm, *n* = 30), long ellipsoid to fusoid, brown when mature, 1-septate, slightly constricted at the septum, guttulate, smooth, thin-walled. **Asexual morph**: Undetermined.

###### Culture characteristics.

Ascospores germinate on PDA within 24 h at room temperature. Germ tubes are produced from the basal or apical cell of the ascospore. Colonies on PDA reach 40 mm in diameter after 2 weeks at 20–25 °C; mycelium superficial and circular; surface rough, dense, white, floccose, and umbonate; reverse white.

###### Material examined.

China • Yunnan Province, Kunming, Xiaokong, on decaying branches of *Betula albosinensis* (*Betulaceae*), 21 December 2019, G.C. Ren, KM09 (HKAS 122745, holotype), ex-type living culture KUNCC 21-0593.

###### GenBank numbers.

ITS: PZ457831; LSU: PZ454099; SSU: PZ487685; *tef*1-α: PZ480920; *rpb*2: PZ480924.

###### Notes.

**Figure 7.**
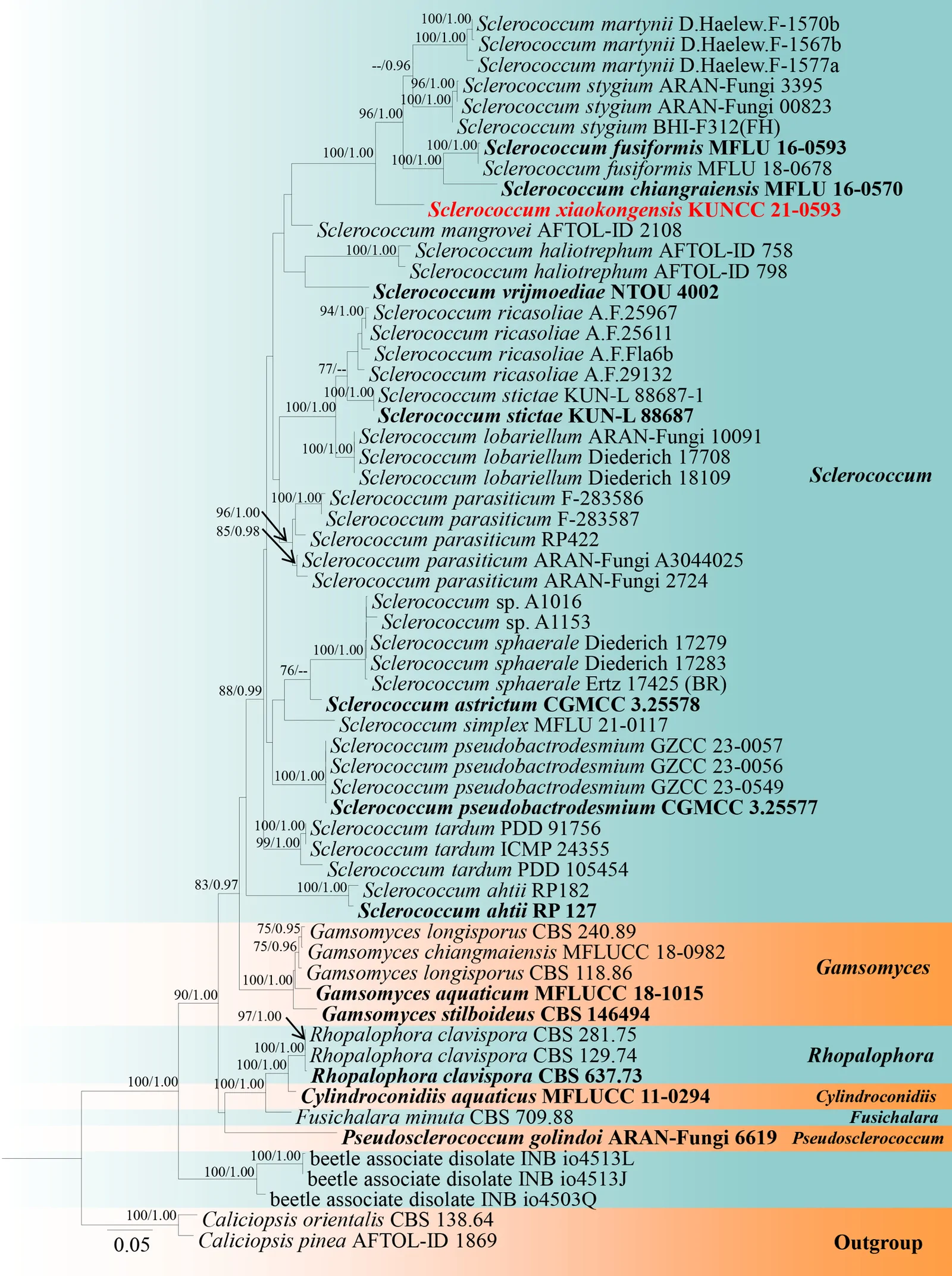
Phylogram generated from ML analysis based on LSU, ITS, and mtSSU sequence data. Related sequences were obtained following Ma et al. (2024) and Meng et al. (2025). Sixty strains are included in the combined analyses, which comprise 2,061 characters for the LSU, ITS, and mtSSU alignments. *Caliciopsis orientalis* (CBS 138.64) and *C. pinea* (AFTOL-ID 1869) were used as the outgroup taxa. The best-scoring RAxML tree with a final likelihood value of –14,037.126149 is presented. The matrix had 889 distinct alignment patterns, with 31.16% undetermined characters or gaps. Estimated base frequencies were as follows: A = 0.289098, C = 0.195072, G = 0.253293, T = 0.262537. Substitution rates were AC = 0.974881, AG = 2.596250, AT = 1.640833, CG = 0.808033, CT = 5.464113, and GT = 1.0000. Gamma distribution shape parameter *α* = 0.255036 and tree length = 2.294224. The tree topology of the ML analysis is similar to that of the Bayesian analysis. Bootstrap values for ML equal to or greater than 70% and posterior probability values greater than 0.95 from the BYPP analysis are labeled at the nodes. Strains of the newly described species are in red, while type strains are in bold.

Multigene phylogenetic analyses based on a concatenated LSU, ITS, and SSU sequence dataset show that the strain KUNCC 21-0593 clusters with *Sclerococcum* species with 100% ML bootstrap support and 1.00 BYPP (Fig. 7) and is closely related to *S. chiangraiensis*, *S. fusiformis*, *S. martynii*, and *S. stygium*. Morphologically, the strain (HKAS 122745, holotype) is distinguished from *S. chiangraiensis* (MFLU 16-0570, holotype) by having smaller ascomata (235–260 × 600–840 μm vs. 450–500 × 1,000–1,500 μm), black apothecia with black margins, and a flat disc, whereas *S. chiangraiensis* (MFLU 16-0570, holotype) has black apothecia with maroon to brown margins and a flat to slightly concave disc (Ekanayaka et al. 2019b). The strain (HKAS 122745, holotype) is distinguished from *S. fusiformis* (MFLU 16-0593, holotype) by having smaller ascomata (235–260 × 600–840 μm vs. 400–1,200 × 200–255 μm), smaller asci (53–74 × 8.8–12 μm vs. 70–80 × 12–18 μm), and smaller ascospores (13–19 × 4–4.9 μm vs. 17–21 × 4–7 μm) (Ekanayaka et al. 2019b). The strain (HKAS 122745, holotype) is distinguished from *S. martynii* (K(M) 264777, holotype) and *S. stygium* (ARAN-Fungi 00823) by having cup-shaped ascomata, whereas *S. martynii* (K(M) 264777, holotype) and *S. stygium* (ARAN-Fungi 00823) have compound ascomata with cespitose branches bearing distal apothecia that are often overlapping or fused (Butler 1940; Haelewaters et al. 2022). Based on both morphological distinctions and its well-supported phylogenetic placement, *Sclerococcum xiaokongense* is introduced as a novel species.

**Figure 8.**
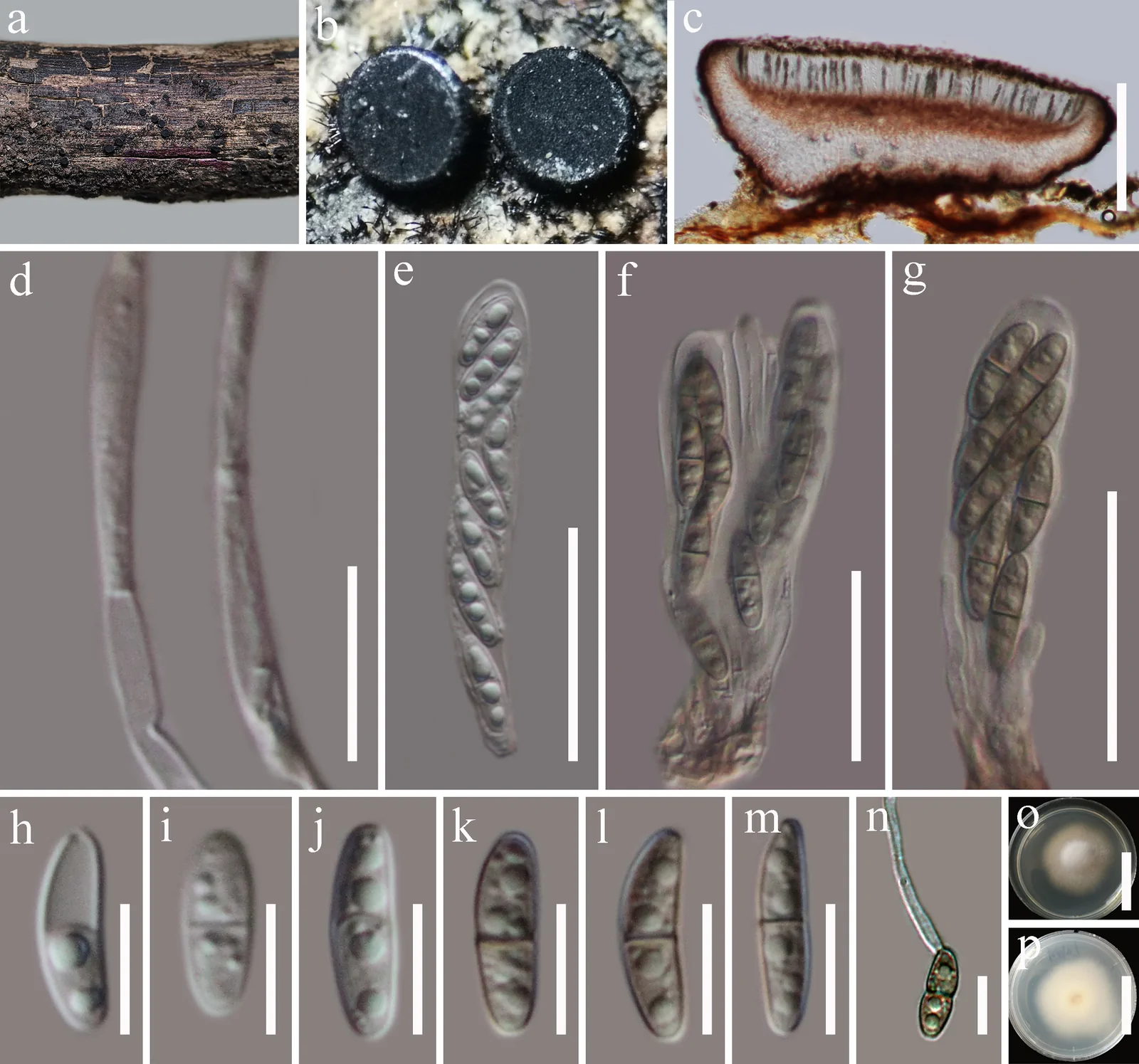
*Sclerococcum xiaokongense* (HKAS 122745, holotype). **a**. Substrate; **b**. Ascomata on wood; **c**. Cross section of an ascoma; **d**. Paraphyses; **e**. Immature ascus; **f, g**. Mature asci; **h–m**. Ascospores; **n**. Germinating ascospore; **p, q**. Colony on PDA, surface, and reverse views. Scale bars: 200 μm (**c**); 10 μm (**d, h–n**); 30 μm (**e–g**); 30 mm (**p, q**).

#### *Leotiomycetes* O.E. Erikss. & Winka, 1997

***Helotiales* Nannf. 1932**

***Lachnaceae* Raitv., Scripta Mycol., Tartu 20: 7 (2004)**

##### Proliferodiscus

Taxon classificationFungiTubeufialesLachnaceae

J.H. Haines & Dumont, Mycologia 75(3): 536 (1983)

505E44E0-689D-5FEE-A914-3CCAAD8C0068

###### Notes.

*Proliferodiscus* was established by Haines and Dumont (1983) to accommodate two species with proliferous hymenial discs, namely *Proliferodiscus inspersus*, which is the type species, and *P. earoleuca*. Species of *Proliferodiscus* are widely distributed worldwide (Luo et al. 2025). Members of the genus have been reported predominantly from wood and bark of various woody hosts (Bien and Damm 2020). Species delimitation within *Proliferodiscus* has traditionally been based on morphological characteristics of the sexual morphs. Currently, only one asexual morph has been described in the genus, namely that of *Proliferodiscus ingens* (Bien and Damm 2020). In the present study, the asexual morph of *Proliferodiscus chiangraiensis* from China is described, representing the second documented asexual morph within the genus.

##### Proliferodiscus chiangraiensis

Taxon classificationFungiTubeufialesLachnaceae

Ekanayaka & K.D. Hyde, Mycosphere 10(1): 403 (2019)

74F89828-C845-5E32-A1EC-A920A39957FE

Index Fungorum: IF556293

Facesoffungi Number: FoF05912

Fig. 10

###### Description.

***Saprobic*** on decaying branches in terrestrial habitats. **Sexual morph**: Undetermined. **Asexual morph**: coelomycetous: ***Conidiomata*** 240–300 µm high × 300–450 µm diam. (x̄ = 260 × 380 µm, *n* = 5), solitary or aggregated, superficial, globose to subglobose, multilocular, black, each locule 76–200 µm high × 70–170 µm diam. (x̄ = 136 × 110 µm, *n* = 5), with a central ostiole. ***Conidiomatal wall*** 10–21 µm wide, composed of an outer layer of brown cells and an inner layer of hyaline cells of ***textura angularis***. ***Conidiophores*** hyaline, smooth-walled, septate, conidiogenous loci formed terminally. ***Conidiogenous cells*** 8–13 × 1–2 μm (x̄ = 10 × 1.4 µm, *n* = 20), enteroblastic, hyaline, smooth-walled, cylindrical, tapering towards apices. ***Conidia*** 2.5–3.3 × 1–1.7 μm (x̄ = 2.8 × 1.4 µm, *n* = 30), cylindrical, straight, hyaline, with both ends rounded.

###### Culture characteristics.

Conidia germinate on PDA within 24 h at room temperature. Germ tubes are produced at the basal region. Colonies on PDA reach 20 mm in diameter after 2 weeks at 20–25 °C; mycelium superficial, circular, fimbriate, and umbonate, pale yellowish at the center and white at the edge, with yellowish granular oil droplets; reverse light brown at the center and white at the edge.

###### Material examined.

China • Yunnan Province, Lancang, lahu Autonomous Prefecture, Hani, on decaying branches of *Cinnamomum glanuliferum* (*Lauraceae*), 20 July 2020, G.C. Ren, LGY24 (HKAS 122736), living culture KUNCC 21-0560.

###### Known distribution and hosts.

China and Thailand, on dead stems and decaying branches of *Cinnamomum glanuliferum* (Ekanayaka et al. 2019a; this study).

###### GenBank numbers.

ITS: PZ457832; LSU: PZ454100; SSU: PZ487686.

###### Notes.

**Figure 9.**
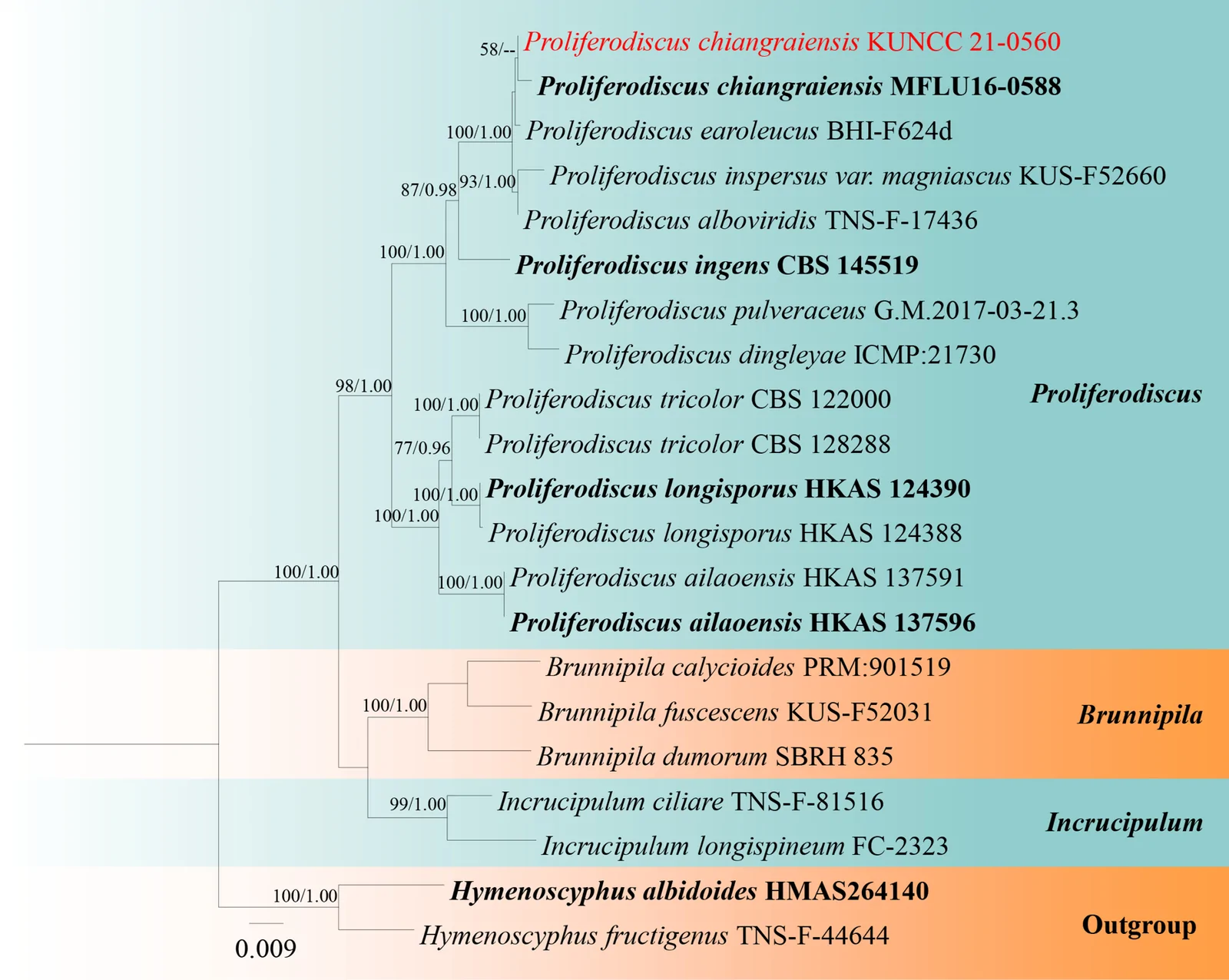
Phylogram generated from ML analysis based on LSU and ITS sequence data. Related sequences were obtained following Luo et al. (2025). Twenty-one strains are included in the combined analyses, which comprise 1,423 characters for the LSU and ITS alignment. *Hymenoscyphus albidoides* (HMAS264140) and *H. fructigenus* (TNS-F-44644) were used as the outgroup taxa. The best-scoring RAxML tree with a final likelihood value of –4,425.646414 is presented. The matrix had 307 distinct alignment patterns, with 20.77% undetermined characters or gaps. Estimated base frequencies were as follows: A = 0.242767, C = 0.223950, G = 0.279807, T = 0.253475. Substitution rates were AC = 0.839962, AG = 1.865002, AT = 1.181724, CG = 0.734813, CT = 4.951913, and GT = 1.0000. Gamma distribution shape parameter *α* = 0.158306 and tree length = 0.423218. The tree topology of the ML analysis is similar to that of the Bayesian analysis. Bootstrap values for ML equal to or greater than 70% and posterior probability values greater than 0.95 from the BYPP analysis are labeled at the nodes. Strains of the newly described species are in red, while type strains are in bold.

In the multigene phylogenetic analyses based on a combined LSU and ITS dataset, the strain KUNCC 21-0560 clustered with the reference strain of *Proliferodiscus chiangraiensis* (MFLU16-0588) (Fig. 9), which was isolated from dead stems in Thailand (Ekanayaka et al. 2019a), although no significant internal statistical support was recovered for this clade. Morphologically, no asexual morph has previously been reported for *Proliferodiscus chiangraiensis* (Ekanayaka et al. 2019a). The characteristics of the strain (HKAS 122736) resemble those of *Proliferodiscus ingens* (GLM-F110834, holotype) in having subglobose, superficial conidiomata; hyaline, smooth-walled conidiophores; enteroblastic, hyaline, cylindrical conidiogenous cells; and hyaline, smooth-walled, aseptate, cylindrical to ellipsoidal conidia (Bien and Damm 2020). Sequence comparison of the ITS region between the strain (KUNCC 21-0560) and *Proliferodiscus chiangraiensis* (MFLU16-0588) showed a 0.89% (5/558 bp, without gaps) base-pair difference in the ITS region and no significant base-pair difference in the LSU region. Based on the phylogenetic analyses, the strain KUNCC 21-0560 is introduced as the first record of the asexual morph of *Proliferodiscus chiangraiensis*.

**Figure 10.**
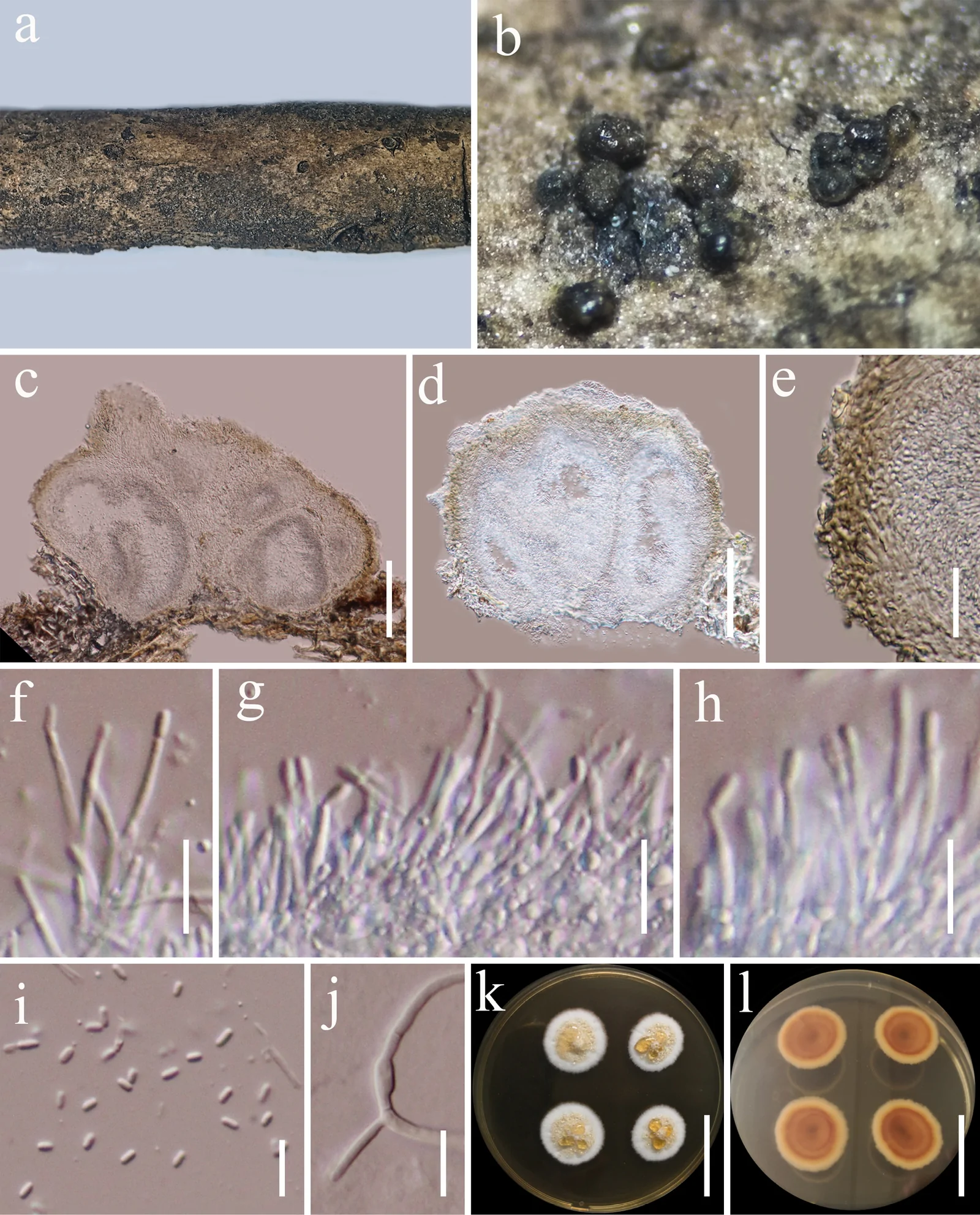
*Proliferodiscus chiangraiensis* (HKAS 122736). **a**. Herbarium specimen; **b**. Conidiomata on substrate; **c, d**. Cross sections of conidiomata; **e**. Conidiomatal wall; **f–h**. Conidiogenous cells lining the inner wall of a conidioma; **i**. Conidia; **j**. Germinated conidium; **k, l**. Colonies on PDA, surface, and reverse views. Scale bars: 100 μm (**c, d**); 20 μm (**e**); 10 μm (**f–j**); 30 mm (**k, l**).

#### *Sordariomycetes* O.E. Erikss. & Winka

***Diaporthales* Nannf**

***Coryneaceae* Corda**

##### Coryneum

Taxon classificationFungiTubeufialesCoryneaceae

Nees, Syst. Pilze (Würzburg): 34 (1816) [1816–17]

0F0074CC-AD87-51A4-A804-144B84C45C47

###### Notes.

*Coryneum* was introduced by Nees von Esenbeck (1816) to accommodate *C. umbonatum* as the type species. The genus is known from both its asexual and sexual morphs. The asexual morph is coelomycetous, characterized by acervular conidiomata, cylindrical, septate conidiophores, cylindrical, annellidic conidiogenous cells, and clavate to subcylindrical, fusiform, or club-shaped, hyaline to brown, distoseptate, guttulate conidia (Jiang et al. 2018; Hongsanan et al. 2025). The sexual morph is characterized by solitary, scattered pseudostromata, globose to subglobose, uniloculate perithecia, long ostiolar necks, 8-spored, unitunicate, clavate to cylindrical asci, and fusiform to ellipsoidal, hyaline to dark brown, 1-septate (occasionally distoseptate), guttulate ascospores (Jiang et al. 2018; Hongsanan et al. 2025; Wang et al. 2025). Currently, 237 *Coryneum* species are listed in Index Fungorum (2026), and only a few species are supported by sequence data in GenBank. Members of *Coryneum* are commonly reported as phytopathogens, saprobes, and soil fungi (Jiang et al. 2019; Hongsanan et al. 2025). Herein, two new species of *Coryneum* from decaying branches in China are introduced.

##### Coryneum lincangense

Taxon classificationFungiTubeufialesCoryneaceae

G.C Ren & W.Y Tian sp. nov.

19A6C812-B318-5E98-84B8-0D8D63BB5D11

Index Fungorum: IF905586

Facesoffungi Number: FoF20024

Fig. 12

###### Etymology.

The specific epithet “*lincangense*” refers to Lincang, where the holotype was collected.

###### Holotype.

HKAS 122753.

###### Description.

***Saprobic*** on decaying branches in terrestrial habitats. **Sexual morph**: Undetermined. **Asexual morph: *Conidiomata*** acervular, 150–210 µm high × 330–350 µm diam. (x̄ = 180 × 340 µm, *n* = 5), globose to subglobose, solitary, superficial to semi-immersed, scattered, velvety, black, with surface tissues composed of thick-walled, brown *textura angularis*. ***Conidiophores*** 17–30 × 3–5 μm (x̄ = 23 × 3.7 µm, *n* = 20), cylindrical, pale brown at the apex, dark brown at the base, smooth, septate, unbranched, emerging from the basal stroma. ***Conidiogenous cells*** 5.6–12.5 × 3–4 μm (x̄ = 8.6 × 3.5 µm, *n* = 20), annellidic, integrated, terminal, cylindrical, truncate apex, pale brown, smooth, exhibiting 1–3 slightly percurrent proliferations. ***Conidia*** 35–54 × 14.5–18 μm (x̄ = 44 × 16.5 µm, *n* = 30), variable in shape, clavate to fusiform, dark brown, smooth-walled, with 5–8 distosepta, guttulate, apical cell with a hyaline tip for fusiform, rounded at apex for clavate, truncate at the base.

###### Culture characteristics.

Conidia germinate on PDA within 24 h at room temperature. Germ tubes are produced from the ends of the conidia. Colonies on PDA reach 45 mm in diameter after 2 weeks at 20–25 °C; mycelium superficial, circular, and fimbriate; surface smooth with an entire margin, umbonate, with a white outer zone and gray center; reverse yellowish at the edge and brown at the center.

###### Material examined.

China • Yunnan Province, Lincang, on decaying branches of *Cinnamomum glanuliferum* (*Lauraceae*), 12 August 2020, G.C. Ren, LC37 (holotype, HKAS 122753), ex-type culture KUNCC 21-0611.

###### GenBank numbers.

ITS: PP663081; LSU: PP663088; SSU: PZ487687; *tef*1-α: PZ480921; *rpb*2: PZ480925.

###### Notes.

**Figure 11.**
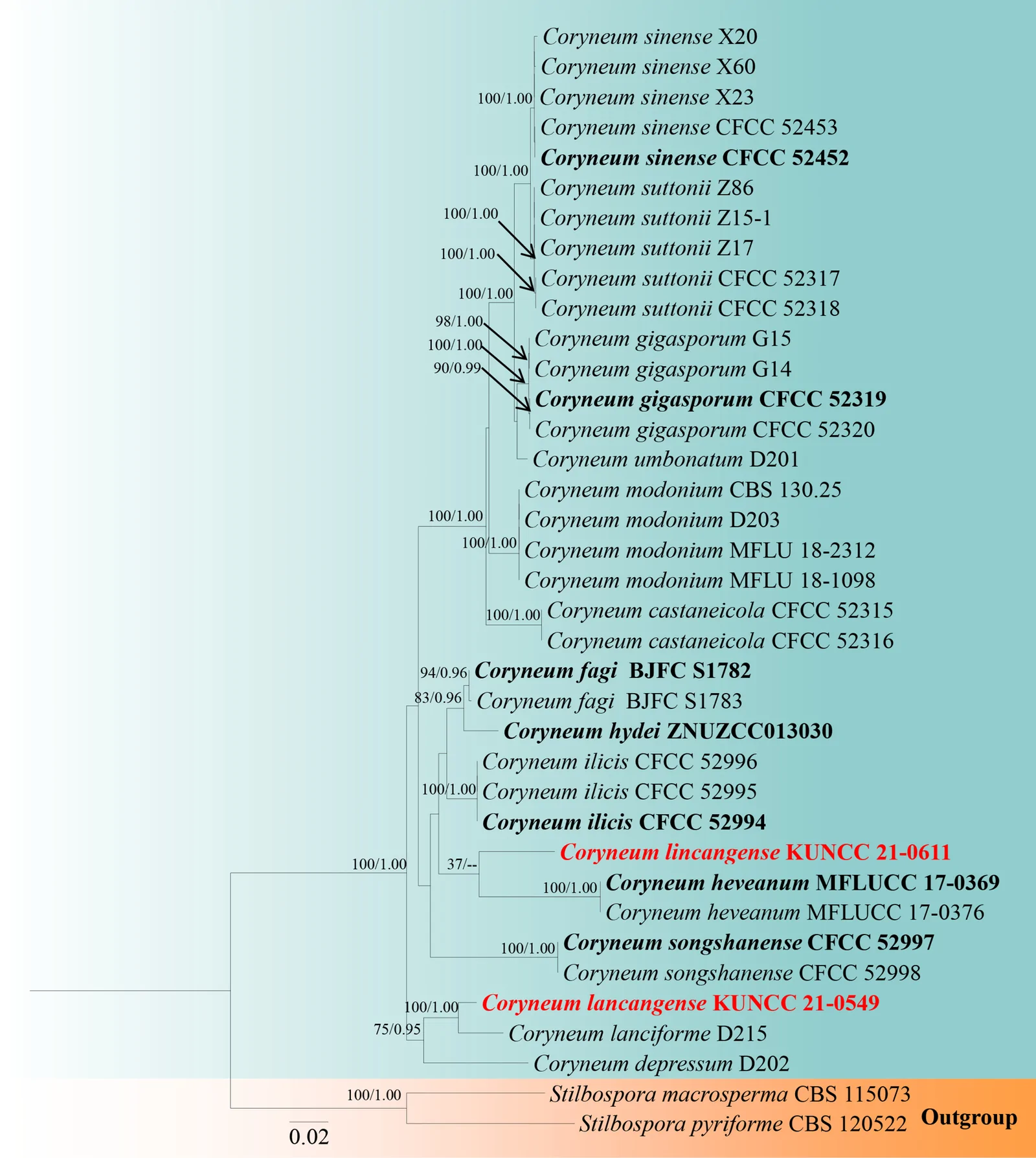
Phylogram generated from ML analysis based on ITS, LSU, *tef*1-α, and *rpb*2 sequence data. Related sequences were obtained following Jiang et al. (2019) and BLAST search results in GenBank. Thirty-seven strains are included in the combined analyses, which comprise 3,788 characters for the ITS, LSU, *tef*1-α, and *rpb*2 alignment. *Stilbospora macrosperma* (CBS 115073) and *S. pyriforme* (CBS 120522) were used as the outgroup taxa. The best-scoring RAxML tree with a final likelihood value of –13,596.559319 is presented. The matrix had 927 distinct alignment patterns, with 27.45% undetermined characters or gaps. Estimated base frequencies were as follows: A = 0.238028, C = 0.273280, G = 0.278322, T = 0.210371. Substitution rates were AC = 1.466103, AG = 2.334780, AT = 1.345806, CG = 1.047633, CT = 6.676255, and GT = 1.0000. Gamma distribution shape parameter *α* = 0.158306 and tree length = 0.423218. The tree topology of the ML analysis is similar to that of the Bayesian analysis. Bootstrap values for ML equal to or greater than 70% and posterior probability values greater than 0.95 from the BYPP analysis are labeled at the nodes. Strains of the newly described species are in red, while type strains are in bold.

Multigene phylogenetic analyses based on a concatenated ITS, LSU, *tef*1-α, and *rpb*2 sequence dataset show that the strain KUNCC 21-0611 clusters with *Coryneum heveanum* (MFLUCC 17-0369, MFLUCC 17-0376) (Fig. 11). Morphologically, *Coryneum lincangense* (HKAS 122753, holotype) is characterized by superficial to semi-immersed, globose to subglobose conidiomata; unbranched conidiophores that are pale brown at the apex and dark brown at the base; and conidia that exhibit either a hyaline apical tip in fusiform forms or rounded apices in clavate forms. In contrast, *Coryneum heveanum* (MFLU 18-0936, holotype) features erumpent conidiomata that emerge through the outer periderm layers of the host, appearing slightly dome-shaped and velvety; conidiophores that are apically pale brown, paler toward the base, and basally branched; and conidia that are rounded or occasionally truncate at the apex and black at the base (Senwanna et al. 2018). *Coryneum heveanum* was found as a saprobe on twigs attached to a tree of *Hevea brasiliensis* in Thailand (Senwanna et al. 2018), whereas *C. lincangense* was found as a saprobe on decayed branches in China. Sequence comparison of the ITS region between *Coryneum lincangense* (KUNCC 21-0611) and *C. heveanum* (MFLUCC 17-0369) showed an 8.4% (44/524 bp, without gaps) base-pair difference in the ITS region and a 1.1% (9/824 bp, without gaps) base-pair difference in the LSU region. Data for *rpb*2 and *tef*1-α are not available for *C. heveanum*, so base-pair differences in these gene regions could not be compared. Based on both morphological distinctions and phylogenetic analyses, *Coryneum lincangense* is introduced as a novel species.

**Figure 12.**
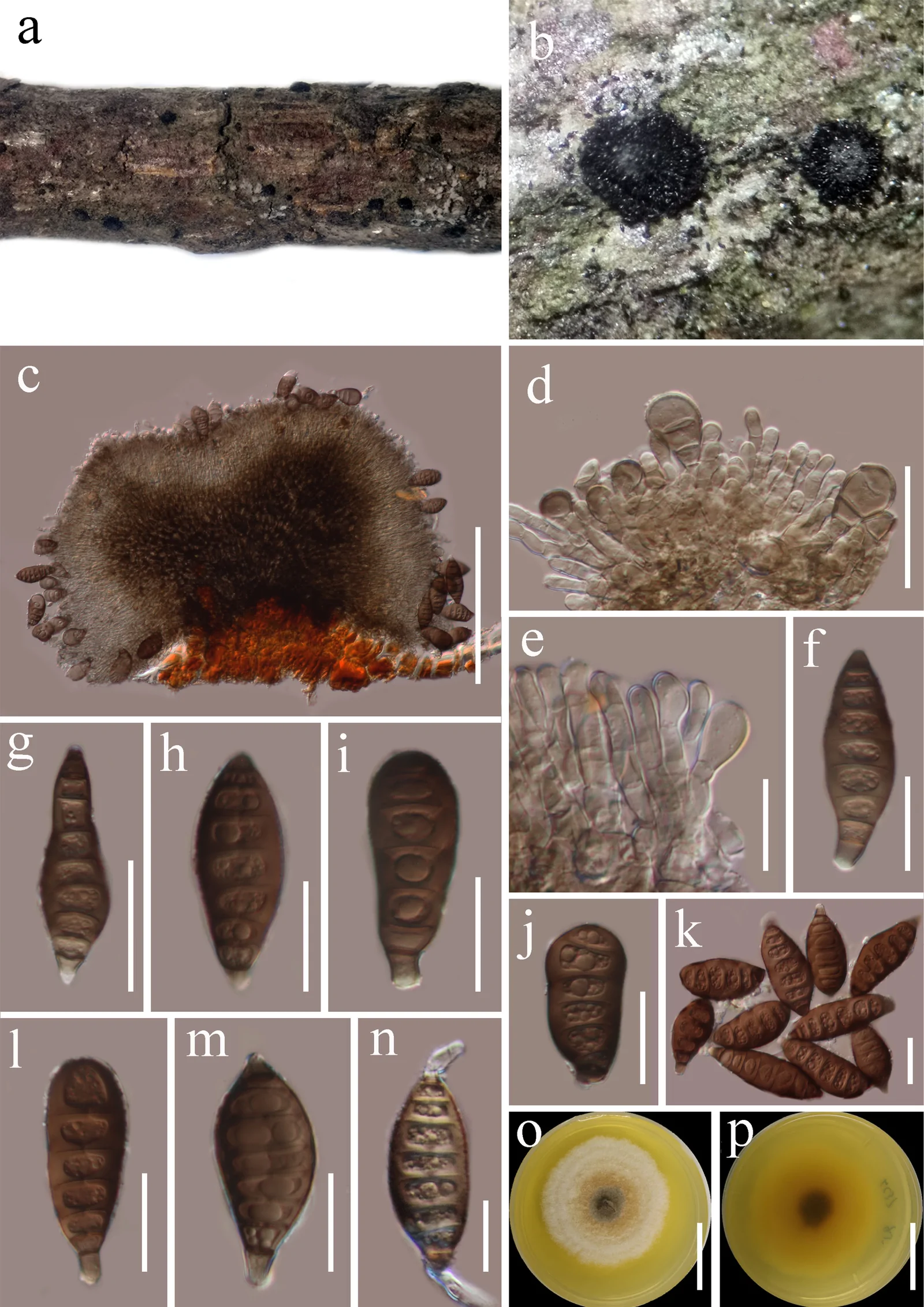
*Coryneum lincangense* (HKAS 122753, holotype). **a, b**. Conidiomata on host surface; **c**. vertical cross section of a conidioma; **d, e**. Conidiophores and conidiogenous cells with conidia; **f–m**. Conidia; **n**. Germinated conidium; **o, p**. Colony on PDA, surface, and reverse views. Scale bars: 150 μm (**c**); 30 μm (**d**); 15 μm (**e**); 20 μm (**f–n**); 30 mm (**o, p**).

##### Coryneum lancangense

Taxon classificationFungiTubeufialesCoryneaceae

G.C Ren & W.Y Tian sp. nov.

C75187A1-0445-5A69-84C4-35BB76C39168

Index Fungorum: IF905587

Facesoffungi Number: FoF20025

Fig. 13

###### Etymology.

The specific epithet “*lancangense*” refers to Lancang, where the holotype was collected.

**Figure 13.**
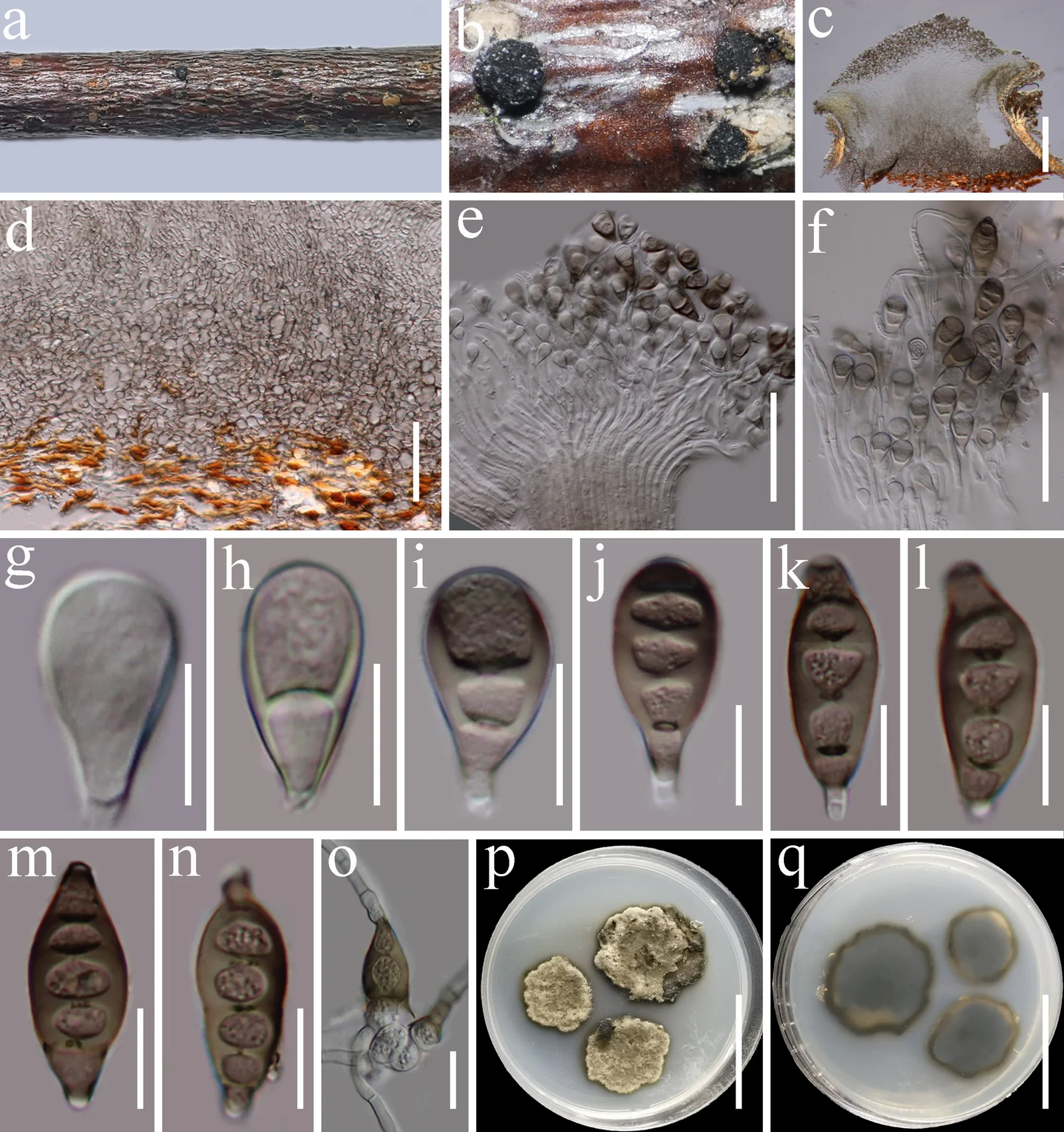
*Coryneum lancangense* (HKAS 122728, holotype). **a, b**. Conidiomata on host surface; **c, d**. Vertical cross sections of a conidioma; **e, f**. Conidiophores and conidiogenous cells with conidia; **g–n**. Conidia; **o**. Germinated conidium; **p, q**. Colonies on PDA, surface, and reverse views. Scale bars: 200 μm (**c**); 50 μm (**d–f**); 15 μm (**g–o**); 20 mm (**p, q**).

###### Holotype.

HKAS 122728.

###### Description.

***Saprobic*** on decaying branches in terrestrial habitats. **Sexual morph**: Undetermined. **Asexual morph: *Conidiomata*** acervular 600–660 µm high × 660–780 µm diam. (x̄ = 625 × 735 µm, *n* = 5), surface tissues above flat or slightly dome-shaped, solitary, semi-immersed to erupting through the host surface, scattered, black, with composed of thick-walled, brown *textura angularis*. ***Conidiophores*** 170–220 × 2.5–4 μm (x̄ = 190 × 3 µm, *n* = 20), cylindrical, hyaline at the apex, pale brown at the base, smooth, septate, unbranched, emerging from the basal stroma. ***Conidiogenous cells*** 20–40 × 2–4 μm (x̄ = 29 × 2.7 µm, *n* = 20), annellidic, integrated, terminal, cylindrical, hyaline, smooth, exhibiting 1–3 slightly percurrent proliferations. ***Conidia*** 27–40 × 13–17 μm (x̄ = 33 × 14.8 µm, *n* = 30), variable in shape, clavate to fusiform, hyaline to pale brown when young, becoming dark brown at maturity, smooth-walled, with 3–5 distosepta, guttulate, apical cell with a hyaline tip for fusiform, rounded at apex for clavate, truncate at the base.

###### Culture characteristics.

Conidia germinate on PDA within 24 h at room temperature. Germ tubes are produced from the ends of the conidia. Colonies on PDA reach 18 mm in diameter after 2 weeks at 20–25 °C; mycelium superficial, circular, and raised; surface rough with an undulate margin, dry, hard, and grayish white; reverse dark brown at the edge and dark gray at the center.

###### Material examined.

China • Yunnan Province, Lancang, Lahu Autonomous Prefecture, Hani, on decaying branches of *Trigonobalanus doichangensis* (*Fagaceae*), 20 July 2020, G.C. Ren, LGY10 (holotype, HKAS 122728), ex-type culture KUNCC 21-0549.

###### GenBank numbers.

ITS: PP663080; LSU: PP663087; SSU: PZ487688; *tef*1-α: PZ480922.

###### Notes.

Multigene phylogenetic analyses based on a concatenated ITS, LSU, *tef*1-α, and *rpb*2 sequence dataset show that the strain KUNCC 21-0549 clusters with *Coryneum lanciforme* (D215) with 100% ML bootstrap support and 1.00 BYPP (Fig. 11). Morphologically, the conidia of *Coryneum lancangense* (HKAS 122728, holotype) (27–40 × 13–17 μm) are comparatively smaller than those of *C. depressum* (D202) (44–53 × 19–23 μm) and *C. lanciforme* (D215) (45–53 × 16–18 μm) (Sutton 1980; Senwanna et al. 2018). *Coryneum depressum* was found on a dead branch of *Quercus* sp. in Germany (Sutton 1980), and *C. lanciforme* was found on branches of *Betula* sp. worldwide (Cannon 2009; Senwanna et al. 2018), whereas *C. lancangense* was found as a saprobe on decayed branches in China. Sequence comparison of the ITS region between *Coryneum lancangense* (KUNCC 21-0549) and *C. lanciforme* (D215) showed a 2.6% (15/573 bp, without gaps) base-pair difference in the ITS region, a 0.84% (7/838 bp, without gaps) base-pair difference in the LSU region, and a 2.7% (20/730 bp, without gaps) base-pair difference in the *tef*1-α region. Data for *rpb*2 are not available for the new species, so base-pair differences in the *rpb*2 region could not be compared. Based on both morphological distinctions and its well-supported phylogenetic placement, *Coryneum lancangense* is introduced as a novel species.

## Discussion

Over the past 8 years, investigations of saprobic microfungi on decaying branches and other woody substrates in Yunnan Province have consistently uncovered previously undocumented taxa (Ren et al. 2020, 2021, 2024; Wanasinghe et al. 2022; Hyde et al. 2024b). In the present study, integrative analyses based on morphology and multigene phylogeny resulted in the identification of five new species and two new records representing four classes of *Ascomycota* (*Dothideomycetes*, *Eurotiomycetes*, *Leotiomycetes*, and *Sordariomycetes*). The recovery of taxonomically diverse fungi from a relatively limited sampling effort highlights the significance of decaying branches as important yet insufficiently explored microhabitats for saprobic fungi. A broader review of fungal discoveries in China during 2000–2020 revealed that the majority of new species have come from southern provinces, with Yunnan alone accounting for more than a quarter of the national total (Wang et al. 2021). Additionally, over 300 novel species have been reported from Yunnan Province in the past 5 years alone (Phookamsak et al. 2024). This disproportionate richness suggests that a vast number of undescribed taxa still await discovery in the region. However, Yunnan’s exceptional biodiversity is increasingly threatened by habitat fragmentation, climate change, and anthropogenic pressures; these pressures pose particular risks to the survival of many fungi (Hongsanan et al. 2023). Thus, fungal taxonomic research should be further strengthened. Such research can not only clarify the fungal resources in Yunnan Province but also provide a basis for biodiversity conservation and ecosystem monitoring.

The introduction of *Anastomitrabeculia yunnanensis* expands the phylogenetic and ecological understanding of the poorly studied family *Anastomitrabeculiaceae*, which currently comprises the single genus *Anastomitrabeculia* (Hyde et al. 2024b). Species of the genus have previously been reported from submerged bamboo and terrestrial woody substrates based on both sexual and asexual morphs (Bhunjun et al. 2021; Ren et al. 2024). The discovery of *A. yunnanensis* from decaying branches in terrestrial habitats further broadens the known ecological distribution of the genus and provides additional morphological and phylogenetic data for this understudied lineage.

In the phylogenetic analyses (Fig. 1), *Camarographium clematidis* clusters within *Anastomitrabeculia*, forming a clade sister to *A. xishuangbannaensis* (KUMCC 21-0585, KUMCC 21-0586), whereas *C. stephensii* (CBS 149168, CPC 41923) forms a separate lineage positioned near species of *Acrocalymmaceae* and *Corynesporascaceae*. *Camarographium* was introduced by Bubák (1916) to accommodate *C. stephensii* as the type species and was placed in *Pleosporales* genera *incertae sedis* (Wijayawardene et al. 2016; Hyde et al. 2024b). Currently, nine epithets of *Camarographium* are listed in Index Fungorum (2026), but only *C. clematidis* and *C. stephensii* have molecular data available in NCBI. *Camarographium clematidis* was introduced by Wijayawardene et al. (2016); however, because germinating spores failed to grow on PDA and MEA, the species was described solely based on its asexual morphology. Later, Du et al. (2025) provided the first molecular data for this species and showed in their phylogenetic analysis that *C. clematidis* clustered with *Anastomitrabeculia didymospora*, although *C. stephensii* was not included in their tree. Based on LSU sequence data, Crous et al. (2022) placed *Camarographium* in *Didymosphaeriaceae*. Morphologically, *A. xishuangbannaensis* and *C. clematidis* share key characters, including globose conidiomata; enteroblastic, phialidic, determinate, discrete, hyaline conidiogenous cells; and muriform, brown conidia. The results suggest that *Camarographium clematidis* should be transferred to *Anastomitrabeculia*; however, this proposal is preliminary, and further fresh collections and molecular data are required to resolve its classification.

*Helicoma* is one of the earliest described helicosporous genera (Lu et al. 2023). The asexual morphs of this genus can be divided into two major morphological types: one is characterized by pleurogenous, helicoid conidia, while the other is characterized by acrogenous, helicoid conidia; the sexual morph is characterized by ascomata (Li et al. 2022). In the phylogenetic analyses based on multilocus sequence data (Fig. 5), the phylogenetic tree of *Helicoma* revealed two highly supported main clades (Clade A and Clade B). However, this division does not fully correspond to the traditional morphological classification based on conidial ontogeny. The high morphological similarity among species within *Helicoma* often leads to confusion, and many historically recorded species lack DNA sequence data; moreover, the absence of a unified and widely accepted taxonomic standard has long resulted in a chaotic classification system (Lu et al. 2018, 2023). In particular, cases exist in which species within *Helicoma* are morphologically highly similar but show considerable divergence in molecular data, indicating that accurate identification of helicosporous fungi must rely on phylogenetic evidence (Lu et al. 2023). Therefore, integrating more morphological, molecular phylogenetic, and chemotaxonomic data in future studies is necessary to comprehensively resolve the long-standing taxonomic challenges in the genus *Helicoma*.

Several taxa reported herein also highlight incomplete knowledge regarding the ecological distribution and life-history connections of many saprobic microfungi. The occurrence of *Dictyocheirospora rotunda* on terrestrial woody substrates suggests that some fungi previously regarded as freshwater-associated may occupy broader ecological niches than currently recognized (Shen et al. 2025). The discovery of two saprobic species of *Coryneum* from decaying branches further indicates that the diversity and ecological roles of the genus in natural woody habitats remain insufficiently documented. Likewise, the discovery of the asexual morph of *Proliferodiscus chiangraiensis* and the introduction of a new sexual species of *Helicoma* indicate that sexual–asexual relationships remain poorly documented in many saprobic microfungi. The introduction of *Sclerococcum xiaokongense* and the new records reported herein further expand the known ecological and geographic occurrence of these fungal groups in China (Meng et al. 2025; this study). Collectively, these findings demonstrate the importance of continued culture-based and integrative taxonomic studies for improving fungal classification and understanding the ecological diversity of poorly studied saprobic microfungi.

## Supplementary Material

XML Treatment for Anastomitrabeculia

XML Treatment for Anastomitrabeculia yunnanensis

XML Treatment for Dictyocheirospora

XML Treatment for Dictyocheirospora rotunda

XML Treatment for Helicoma

XML Treatment for Helicoma nixiense

XML Treatment for Sclerococcum

XML Treatment for Sclerococcum xiaokongense

XML Treatment for Proliferodiscus

XML Treatment for Proliferodiscus chiangraiensis

XML Treatment for Coryneum

XML Treatment for Coryneum lincangense

XML Treatment for Coryneum lancangense

## Additional information

### Artificial Intelligence (AI) use

The authors accept full responsibility for the content of the manuscript, including the disclosure of any use of AI.

No AI tools were used in the preparation of this manuscript.

### Author ORCIDs

G.-C. Ren https://orcid.org/0000-0001-9923-2626

W.-H. Chen https://orcid.org/0000-0001-7240-6841

H. Gui https://orcid.org/0000-0002-0946-1589

### Data availability

All of the data that support the findings of this study are available in the main text.
